# Sleep loss potentiates Th17‐cell pathogenicity and promotes autoimmune uveitis

**DOI:** 10.1002/ctm2.1250

**Published:** 2023-05-02

**Authors:** Xiuxing Liu, Yuhan Su, Zhaohao Huang, Jianjie Lv, Chenyang Gu, Zhuang Li, Tianyu Tao, Yidan Liu, Qi Jiang, Runping Duan, Binyao Chen, Rong Ju, Xianggui Wang, Yingfeng Zheng, Wenru Su

**Affiliations:** ^1^ State Key Laboratory of Ophthalmology Zhongshan Ophthalmic Center Sun Yat‐sen University Guangdong Provincial Key Laboratory of Ophthalmology and Visual Science Guangzhou P. R. China; ^2^ Department of Clinical Medicine Zhongshan School of Medicine Sun Yat‐Sen University Guangzhou P. R. China; ^3^ Eye Center of Xiangya Hospital Central South University Changsha P. R. China; ^4^ Hunan Key Laboratory of Ophthalmology Xiangya Hospital, Central South University Changsha P. R. China

**Keywords:** autoimmune disease, GM‐CSF, mass cytometry, myeloid cells, single‐cell RNA sequencing, sleep loss, Th17 cells

## Abstract

**Background:**

Sleep loss (SL) is a health issue associated with the higher risk of autoimmune and inflammatory disorders. However, the connection between SL, the immune system, and autoimmune diseases remains unknown.

**Methods:**

We conducted mass cytometry, single‐cell RNA sequencing, and flow cytometry to analyze how SL influences immune system and autoimmune disease development. Peripheral blood mononuclear cells from six healthy subjects before and after SL were collected and analyzed by mass cytometry experiments and subsequent bioinformatic analysis to identify the effects of SL on human immune system. Sleep deprivation and experimental autoimmune uveitis (EAU) mice model were constructed, and scRNA‐seq data from mice cervical draining lymph nodes were generated to explore how SL influences EAU development and related autoimmune responses.

**Results:**

We found compositional and functional changes in human and mouse immune cells after SL, especially in effector CD4^+^ T and myeloid cells. SL upregulated serum GM‐CSF levels in healthy individuals and in patients with SL‐induced recurrent uveitis. Experiments in mice undergoing SL or EAU demonstrated that SL could aggravate autoimmune disorders by inducing pathological immune cell activation, upregulating inflammatory pathways, and promoting intercellular communication. Furthermore, we found that SL promoted Th17 differentiation, pathogenicity, and myeloid cells activation through the IL‐23Th17GM‐CSF feedback mechanism, thus promoting EAU development. Lastly, an anti‐GM‐CSF treatment rescued SL‐induced EAU aggravation and pathological immune response.

**Conclusions:**

SL promoted Th17 cells pathogenicity and autoimmune uveitis development, especially through the interaction between Th17 and myeloid cells involving GM‐CSF signaling, providing possible therapeutic targets for the SL‐related pathological disorders.

## Graphical abstract

**The IL‐23–Th17–GM‐CSF positive feedback loop drives aggravated autoimmune uveitis after sleep loss**.

SL increases GM‐CSF levels in healthy individuals, patients with SL‐induced recurrent uveitis and mice with experimental autoimmune uveitis (EAU). SL enhances the interaction between Th17 and APC. Th17‐derived GM‐CSF and APC‐derived IL‐23 construct a positive feedback loop to maintain Th17 pathogenicity. SL enhances Th17 pathogenicity through the IL‐23–‐Th17–GM‐CSF feedback mechanism, ultimately leading to AU development.

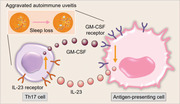

## 1 INTRODUCTION

Sleep is indispensable for maintaining homeostasis.^1^ However, shortened sleep duration and worsened sleep quality are common, resulting in the detrimental health effects that accompany sleep loss (SL).^2^ SL is associated with abnormal hormone secretion, neurocognitive function and academic performance.^3^, ^4^ Moreover, SL is related to a higher risk of several disorders including diabetes, cardio‐metabolic diseases, tumour incidence and SARS‐CoV‐2 infection.^5^, ^6^, ^7^ SL can induce changes in the composition and function of immune cells.^8^ Our previous study has constructed an immune atlas of poor sleep using single‐cell techniques, providing an extensive description of how SL influences the immune cells and demonstrating SL is a predisposing factor for immune‐related diseases.^9^

Sleep disturbances have been noted in autoimmune disorders, such as rheumatoid arthritis (RA), systemic lupus erythematosus (SLE) and Behçet's disease.^10^ Additionally, patients with non‐apnoea sleep disorders had significantly higher odds of developing autoimmune diseases including RA, SLE and ankylosing spondylitis.^11^ Patients with an increased SLE risk self‐reported short sleep duration prior to SLE development.^12^ More importantly, clinical data indicate that short sleep duration (<7 h a day) increases the risk of recurrent autoimmune diseases, increases medication use and leads to socioeconomic burdens and poor disease outcomes.^13^ Although these studies show a positive correlation between SL and autoimmune disease, mechanisms postulated to explain how SL affects the autoimmune pathogenesis remain ambiguous.

Autoimmune uveitis (AU) is an autoimmune disease occurring in the central nervous system (CNS). It features intraocular inflammation and is a major cause of visual impairment, causing in 5 to 25% of legal blindness.^14^ AU's pathology can be researched using experimental autoimmune uveitis (EAU), the most commonly used animal model for AU.^15^ Secondary lymphoid organs, like lymph nodes (LNs), are vital for the adaptive immune response and autoimmune processes. As the key sites that promote the interaction among multiple immune cells, LNs mediate antigen presentation processes and immune cell activation.^16^ Moreover, aberrant immune cell activation and function, especially the interactions of pathological T‐helper 17 cells (Th17) and antigen‐presenting myeloid cells in LNs, are known to initiate and promote autoimmune disorders.^17^, ^18^, ^19^ Cervical draining LNs (CDLNs) are the major draining LNs of the CNS, facilitating the drainage of antigens and immune cells.^20^ Antigen‐presenting myeloid cells and autoreactive lymphocytes then react with the retinal antigens, drain them and recruit inflammatory cells across the blood‐eye barrier, thus, causing inflammatory histopathological damage.^21^ Removing CDLNs by cervical lymphadenectomy can reduce the onset and progression of experimental CNS disease, indicating the importance of CDLNs in autoimmune diseases.^22^ It's reported that SL altered the number of lymphocytes and neutrophils in LNs.^23^ However, previous research has not connected how SL influences the immune responses in CDLNs, contributing to AU and EAU aetiology.

High‐throughput single‐cell technology is an effective tool for understanding immune cell heterogeneity, their intricate cellular interaction and their role in autoimmune pathogenesis.^24^ In this study, by using single‐cell mass cytometry by time of flight (CyTOF) and single‐cell RNA sequencing (scRNA‐seq), we map the immune cell atlas of SL and elucidated the effect of SL on the immune cells and autoimmune‐associated responses. SL induced compositional and functional changes in human and mouse immune cells, especially in effector CD4^+^ TCs and myeloid cells. In addition, SL exacerbated disease symptoms, pathological immune responses and enhanced intercellular communication in EAU mice. Moreover, SL promoted GM‐CSF secretion, the activation and chemotaxis of myeloid cells and the differentiation and pathogenicity of Th17 cells, through IL‐23–Th17–GM‐CSF feedback, which can be interrupted by targeted inhibition of GM‐CSF.

## 2 RESULTS

### 2.1 SL reconstitutes the circulating cellular populations in human blood

**FIGURE 1:**
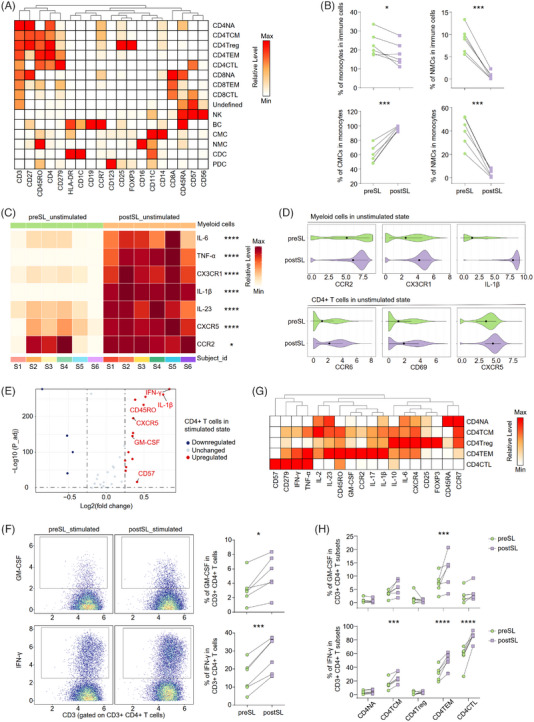
CyTOF validated the human blood immune dysregulation after SL. (A) The heatmap showing the mean expression levels of type markers used for FlowSOM clustering. Colour scheme is based on *z*‐score distribution from minimum (white) to maximum (red). (B) The percentage of monocytes, CMCs and NMCs between pre‐SL and post‐SL groups (n = 6/group). Significance was determined using two‐tailed paired *t*‐test; *p < .05, ***p < .001. (C) The heatmap showing the relative levels of cytokines and markers in myeloid cells under unstimulated condition. Colour scheme is based on *z*‐score distribution from minimum (white) to maximum (red). Significance was determined using the ‘diffcyt‐DS‐GLMM’ method as implemented in the ‘diffcyt’ function of diffcyt R package; *p < .05, ****p < .0001. (D) The violin plot showing the expression of selected markers in myeloid cells (top) and CD4^+^ TCs (bottom) under unstimulated condition between preSL and postSL groups. (E) The volcano plot showing the downregulated and upregulated markers in postSL/preSL comparison in CD4^+^ TCs under stimulated condition. (F) The percentage of GM‐CSF^+^ cells (top) and in IFN‐γ^+^ cells (bottom) in CD3^+^CD4^+^ T cells measured by CyTOF. Scatter charts showing the differences between the two groups derived from CyTOF data (n = 6/group). Significance was determined using two‐tailed paired *t*‐test; *p < .05, ***p < .001. (G) The heatmap showing the relative levels of cytokines and markers in CD4^+^ TC subsets. Colour scheme is based on *z*‐score distribution from minimum (white) to maximum (red). (H) Scatter charts showing proportions of GM‐CSF^+^ cells (top) and in IFN‐γ^+^ cells (bottom) in each T‐cell subset between the two groups derived from CyTOF data (n = 6/group). Significance was determined using two‐tailed paired *t*‐test; ***p < .001, ****p < .0001. The full names of the subsets are as follows: CD4NA, CD4^+^ naive T cell; CD4TCM, central memory CD4*^+^* T cell; CD4Treg, regulatory CD4^+^ T cell; CD4TEM, effector memory CD4^+^ T cell; CD4CTL, cytotoxic CD4^+^ T cell; CD8NA, CD8^+^ naive T cell; CD8TEM, effector memory CD8^+^ T cell; CD8CTL, cytotoxic CD8^+^ T cell; undefined, undefined T cells; NK, natural killer cell; BC, B cell; CMC, classical monocyte; NMC, non‐classical monocyte; CDC, conventional dendritic cell; PDC, plasmacytoid dendritic cell.

To identify the effects of SL on the human immune system, we collected peripheral blood mononuclear cells (PBMCs) from six healthy subjects before and after SL. PBMCs were partly stimulated by stimulation cocktails in an antigen‐independent manner, thus, were divided into four groups: preSL_unstimulated, postSL_unstimulated, preSL_stimulated and postSL_stimulated. We performed CyTOF experiments and the subsequent bioinformatics analysis to map the immune cells based on surface markers and intracellular cytokines with single‐cell resolution^25^ (Supporting Information Table S3). We annotated the FlowSOM‐defined nodes into classical immune cell types, including CD4^+^ naïve (CD4NA), central memory, regulatory, effector memory (CD4TEM) and cytotoxic T cells (CD4CTL), CD8^+^ naïve (CD8NA), effector memory, and cytotoxic T cells (TCs), natural killer cells, B cells (BCs), classical monocytes (CMCs), non‐classical monocytes, conventional dendrite cells (CDCs) and plasmacytoid dendrite cells (Figure 1A, Supporting Information Figure S1A,B) based on previous studies.^26^, ^27^

We explored the effects of SL on cell composition in the unstimulated state because stimulation affected myeloid cell signals.^26^ After SL, we validated that monocyte decreased, which was the main component of myeloid cells (Figure 1B). Interestingly, SL also influenced inflammatory monocyte subsets composition, indicated by the increase of CMC and decrease of non‐classical monocytes (Figure 1B). Moreover, we validated that SL promoted the secretion of inflammatory cytokines by myeloid cells including IL‐1β, IL‐23, IL‐6 and TNF‐α. Similar upregulation was found in the expression of chemotaxis‐associated receptors including CCR2 and CX3CR1 (Figure 1C,D). Thus, SL may induce the inflammatory activation and migration of myeloid cells.

CD4^+^ TCs are important cells involved in adaptive immune system.^16^, ^28^ Post‐SL groups had increase of CCR6‐, CXCR5*‐*, CD45RO‐ and CD69‐expressing CD4^+^ TCs (Figure 1D, Supporting Information Figure S1C), indicating SL promoted the differentiation and activation of CD4^+^ TCs. Next, we validated the effects of SL following cell stimulation. SL increased the memory marker CD45RO, the effector markers CXCR5 and CD57, and the pro‐inflammatory cytokines GM‐CSF, IFN‐γ and IL‐1β (Figure 1E, Supporting Information Figure S1D). The upregulation of inflammatory cytokines was confirmed by the manual gating analysis (Figure 1F, Supporting Information Figure S1E). Then, CD4^+^ TCs were separated into CD4NA, central memory, regulatory, CD4TEM and CD4CTL based on FlowSOM nodes (Figure 1G). We found that GM‐CSF expression was mainly from CD4TEM and IFN‐γ was from CD4CTL (Figure 1G,H). Thus, SL induced inflammatory cytokines associated with autoimmunity in CD4^+^ TCs.

Collectively, SL induces changes in circulating immune subsets, especially inflammatory TCs and myeloid cells. Moreover, SL promoted autoimmune‐associated protein and increased cytokine expression, including GM‐CSF, IFN‐γ and IL‐1β, in effector CD4^+^ TCs.

### 2.2 SL induces autoimmune‐associated changes in mouse LNs

The instruction of SL‐related comprehensive human immune landscape has demonstrated the interaction between SL and human autoimmunity. Given the importance of CDLNs in the development of autoimmune diseases, it is essential to systematically investigate the effects of SL on mouse CDLNs. Mice subjected to SL underwent three rounds of sleep deprivation. In addition, we constructed EAU by using the commonly used protein, retinal antigen inter‐photoreceptor retinoid‐binding protein 1−20 (IRBP_1−20_), as well as the complete Freund's adjuvant and pertussis toxin (PTX).^29^ To rule out the adjuvant‐associated influences, one group of mice was merely injected with complete Freund's adjuvant and PTX, named as control group of IRBP_1−20_‐immunized EAU mice. The blank mice and IRBP_1−20_‐immunized EAU mice undergoing SL were divided into SL and SL EAU (SU) groups, respectively. On day 14 after immunization, scRNA‐seq data were generated from the single‐cell suspensions from CDLNs of the above mice (Supporting Information Figure S2A).

We first compared the immune profiles between SL and blank mice to probe the effects of SL on the immune cells of normal blank mice. After performing unsupervised clustering analysis, we identified seven major immune cell subsets using canonical lineage markers including CD4^+^ TCs (CD4), cytotoxic cells (CYTO), mitotic TCs (MITO), BCs, plasmacytoid dendrite cells, myeloid and TBC (expressed both T and BC markers) (Supporting Information Figure S2B,C). As shown in Supporting Information Figure S2D, CD4, CYTO and BCs were the major cellular constituents of CDLNs. Similar to the human data, SL altered the immune cell proportion in mouse CDLNs.

**FIGURE 2:**
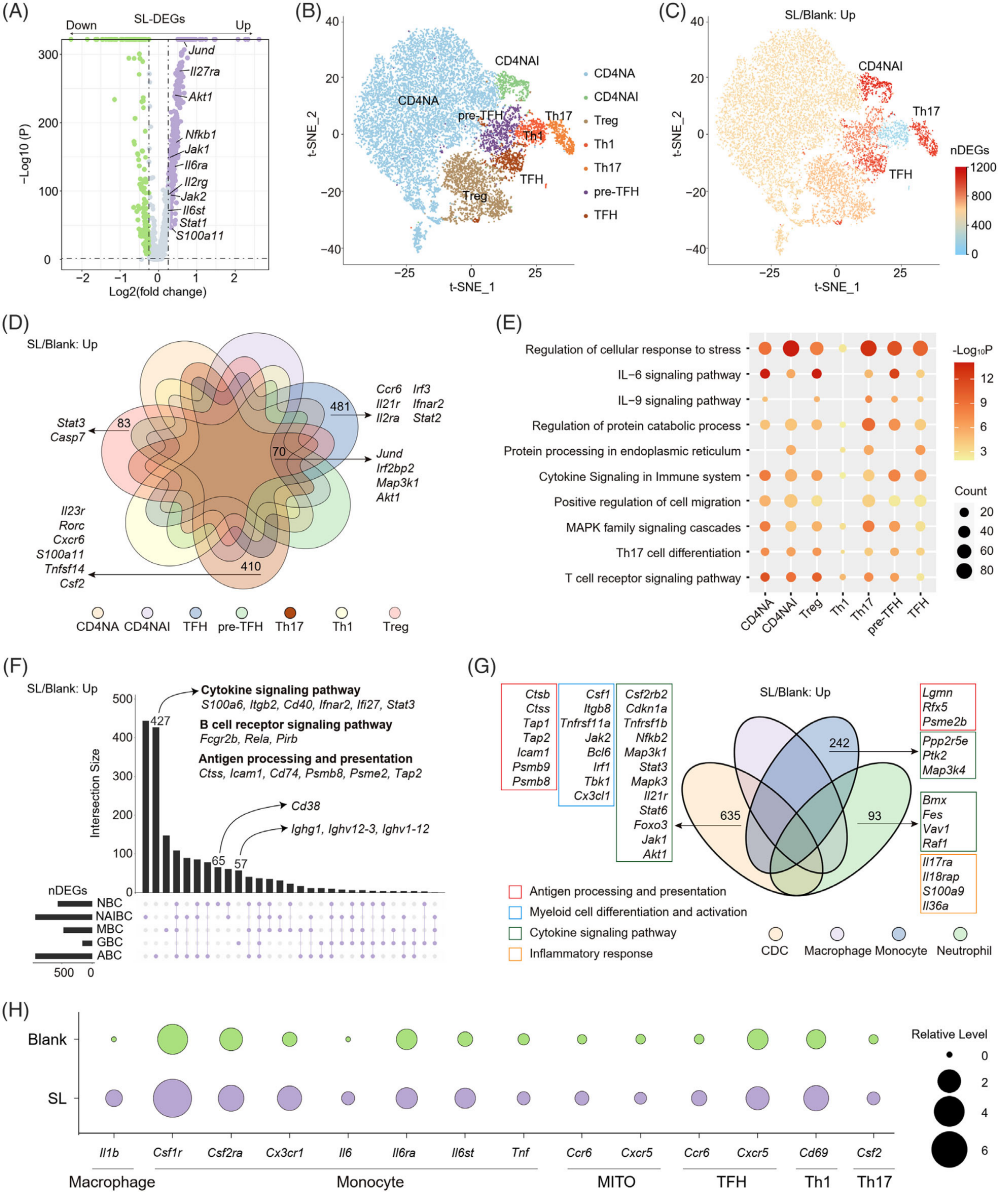
SL induced autoimmune‐associated changes in mice CDLNs. (A) Volcano plot showing the upregulated or downregulated SL‐DEGs in all CDLNs cells. (B) t‐SNE plot showing the CD4^+^ TC subsets of CDLNs in scRNA‐seq. (C) t‐SNE plot showing the number of upregulated SL‐DEGs among CD4^+^ TC subsets. (D) Venn diagram showing the interactions of upregulated SL‐DEGs among CD4^+^ TC subsets. (E) Representative GO biological process and pathways enriched in upregulated SL‐DEGs among CD4^+^ TC subsets. (F) UpSet plot showing the interactions of upregulated SL‐DEGs among BC subsets. (G) Venn diagram showing the interactions of upregulated SL‐DEGs among myeloid subsets. (H) The dot plot showing the relative levels of genes between two groups. The full names of the subsets are as follows: NBC, naive B cell; NAIBC, naive Isg15^+^ B cell; MBC, memory B cell; ABC, age/autoimmune‐associated B cell; GBC, germinal B cell; CD4NA, CD4^+^ naive T cell; CD4NAI, CD4^+^ naive Isg15^+^ T cell; Treg, regulatory CD4^+^ T cell; Th1, T‐helper 1 cell; Th17, T‐helper 17 cell; TFH, Bcl6^high^ S1pr1^−^ T follicular helper cells; pre‐TFH, Bcl6^med^ S1pr1^+^ TFH precursor; CDC, conventional dendritic cell.

We conducted an analysis of differentially expressed genes (DEGs) between SL and blank mice (named SL‐DEGs). Notably, SL elevated genes associated with JAK‐STAT signalling (*Jak2*, *Jak1*, *Stat1*, *Il2rg*, *Il27ra*, *Il6st* and *Il6ra*) and inflammatory responses (*Jund*, *Akt1*, *Nfkb1 and S100a11*) (Figure 2A). To determine the importance of these transcriptional patterns, we conducted Gene Ontology (GO) and pathway enrichment analyses with the upregulated SL‐DEGs. SL increased cytokine signalling and cell activation in MITO cells. In addition, MAPK and IL‐6 signalling increased in CD4^+^ TCs (Supporting Information Figure S2E). Using a Venn diagram of upregulated SL‐DEGs, we found subtype‐specific SL‐upregulated expression patterns, including BC activation‐related genes (*Cd38*, *Ifi209* and *Bank1*) in BCs, and an aging‐related gene (*Cdkn2b*) in myeloid cells (Supporting Information Figure S2F). In MITO, the proliferating T‐cell subset, several genes related to migration (*Ccr6*, *Ccr8* and *Cxcr6*) and interferon signalling (*Ifnar1*, *Ifi27*) were increased after SL. In addition, SL increased inflammation‐related genes *Nfkb1*, *Irf2bp2* and *Itgb2* in five subsets, and *Jund*, *Map3k1* and *Il27ra* in six subsets (Supporting Information Figure S2F). These results indicate SL alters the immune environment of CDLNs and induces inflammatory and autoimmune states.

### 2.3 SL results in compositional and functional alterations in immune sub‐populations of mouse CDLNs

Lymphocytes (containing TCs and BCs) and myeloid cells are the main cellular components of CDLNs, which represent crucial roles in antigen immune responses. We next explored whether the SL‐induced impact on autoimmunity was restricted to a specific lymphocyte or myeloid subset. BCs were re‐clustered into six subsets: naïve BC, *Isg15* ^+^ naïve BC, memory BC, plasma cells, *Fcrl5* ^+^ *Zeb2* ^+^ age/autoimmune‐associated BC (ABCs),^30^, ^31^, ^32^ and germinal BC. CYTO cells were redefined into four subsets: natural killer cells, CD8NA, *Isg15* ^+^ naïve CD8+ (CD8NAI) and CD8^+^ cytotoxic TCs (Supporting Information Figure S3A,B). Additionally, we identified four myeloid subsets: *Tbc1d4* ^+^ *Fscn1* ^+^ CDC, *Apoe* ^+^ *C1qb* ^+^ macrophage, *Lyz2* ^+^ *Ccr2* ^+^ monocyte and *Mmp9* ^+^ *Retnlg* ^+^ neutrophil (Supporting Information Figure S3C). We separated CD4^+^ TCs into seven subsets: CD4NA, *Isg15* ^+^ naïve CD4^+^ (CD4NAI), Treg, T helper 1 cells (Th1), Th17, Bcl6^high^ S1pr1^−^ T follicular helper cells (TFH) and Bcl6^med^ S1pr1^+^ TFH precursor (pre‐TFH)^33^ (Figure 2B, Supporting Information Figure S3D).

CD4NA dominated the CD4^+^ TCs compartments. SL affected the constitution of CD4^+^ subsets, indicating dysregulated immunity (Supporting Information Figure S3E). Th17 cells are actively involved in AU and EAU among all CD4 subsets.^34^ A *t*‐SNE plot with the projected numbers of upregulated SL‐DEGs exhibited prominent transcriptomic changes in the Th17 subset (Figure 2C). Next, we constructed a Venn diagram with the upregulated SL‐DEGs to explore cell‐specific patterns. Th17 differentiation (*Il23r*, *Rorc and Cxcr6*) and pathogenicity genes (*S100a11, Tnfsf14*) were upregulated (Figure 2D). Notably, SL increased the level of GM‐CSF gene *Csf2* in Th17, while the levels of *Ifng* were not altered after SL (Figure 2D, Supporting Information Figure S3F), suggesting the potential role of GM‐CSF in SL‐induced disorders. Additionally, subtype‐specific patterns were also detected, including genes related to T‐cell differentiation (*Ccr6*, *Il21r* and *Il2ra*) and interferon signalling (*Irf3*, *Ifnar2* and *Stat2*) in TFH. In Treg, SL increased the apoptosis‐related genes *Stat3* and *Casp7*. SL downregulated the level of genes associated with oxidative phosphorylation (*mt‐Atp8*, *mt‐Nd1* and *mt‐Co3*) and anti‐apoptosis process (*Bcl2* and *Il7r)*. The genes related to the differentiation and regulatory functions of Treg, like *Ikzf2*, *Ctla4* and *Il2rb*, were also downregulated (Supporting Information Figure S3G). These results suggest that SL induced the inflammatory damage and the immune dyshomeostasis of Treg. In addition, the inflammation‐related genes (*Jund*, *Map3k1* and *Akt1*) were elevated in at least six CD4 subsets (Figure 2D). Functional analysis of upregulated SL‐DEGs revealed upregulated cytokine, MAPK signalling pathways and protein processing, especially in Th17 cells (Figure 2E), indicating an association between SL and Th17‐cell activation. Moreover, we discovered that the IL‐6 signalling pathway was overrepresented in several subsets, including CD4NA and Treg (Figure 2E), corresponding to cellular immune activation. These results indicate that SL induces changes in different CD4^+^ subsets, specifically in Th17 activation.

BCs are another lymphocyte subset that is closely related to adaptive immunity and autoimmune response. Based on an UpSet plot of upregulated SL‐DEGs among BC subsets, we found that SL strongly affects ABCs. Moreover, SL induced upregulation of several genes related to the cytokine signalling pathway, BC receptor signalling pathway and antigen processing and presentation in ABCs (Figure 2F). We also found subtype‐specific expression patterns, including *Cd38* in naïve BCs, and *Ighg1*, *Ighv12‐3* and *Ighv1‐12* in germinal BCs (Figure 2F).

Myeloid cells, including DCs and monocytes, are components of the innate immune system that promote antigen presentation and inflammatory process.^35^ Among myeloid subsets, the CDC was most affected by SL with the greatest number of SL‐DEG (Supporting Information Figure S3H). We generated a Venn diagram and conducted GO analysis of DEGs to probe the functional changes in myeloid subsets. SL induces upregulation in antigen processing and presentation, myeloid cell differentiation and activation and cytokine signalling pathways. These pathways were most evident in the CDC subset (Figure 2G). Notably, SL increased the level of GM‐CSF receptor *Csf2rb2*, which is involved in the maturation and activation of CDC. Additionally, genes related to antigen processing and presentation were also upregulated in monocytes, whereas cytokine signalling pathways were enriched in both monocytes and neutrophils. Several genes associated with inflammatory responses (*Il17ra*, *Il18rap*, *S100a9* and *Il36a*) were upregulated only in neutrophils (Figure 2G).

Given our findings in human samples, we further analysed whether the SL induced immune alterations in mice were consistent. We found commonly upregulated expression of genes in immune cells of humans and mice, including *Il1b* in macrophages, genes related to GM‐CSF and IL‐6 signalling pathway in monocytes. For CD4^+^ TCs, SL increased *Csf2* in Th17, *Ccr6*, *Cxcr5* in MITO and TFH, and *Cd69* in Th1 (Figure 2H). Our results suggest that SL promotes CD4^+^ TCs, BCs and myeloid cell activation and function, promoting inflammatory responses with a strong autoimmune predisposition.

### 2.4 SL promotes EAU development and enhances the pathological immune responses in EAU mice

**FIGURE 3:**
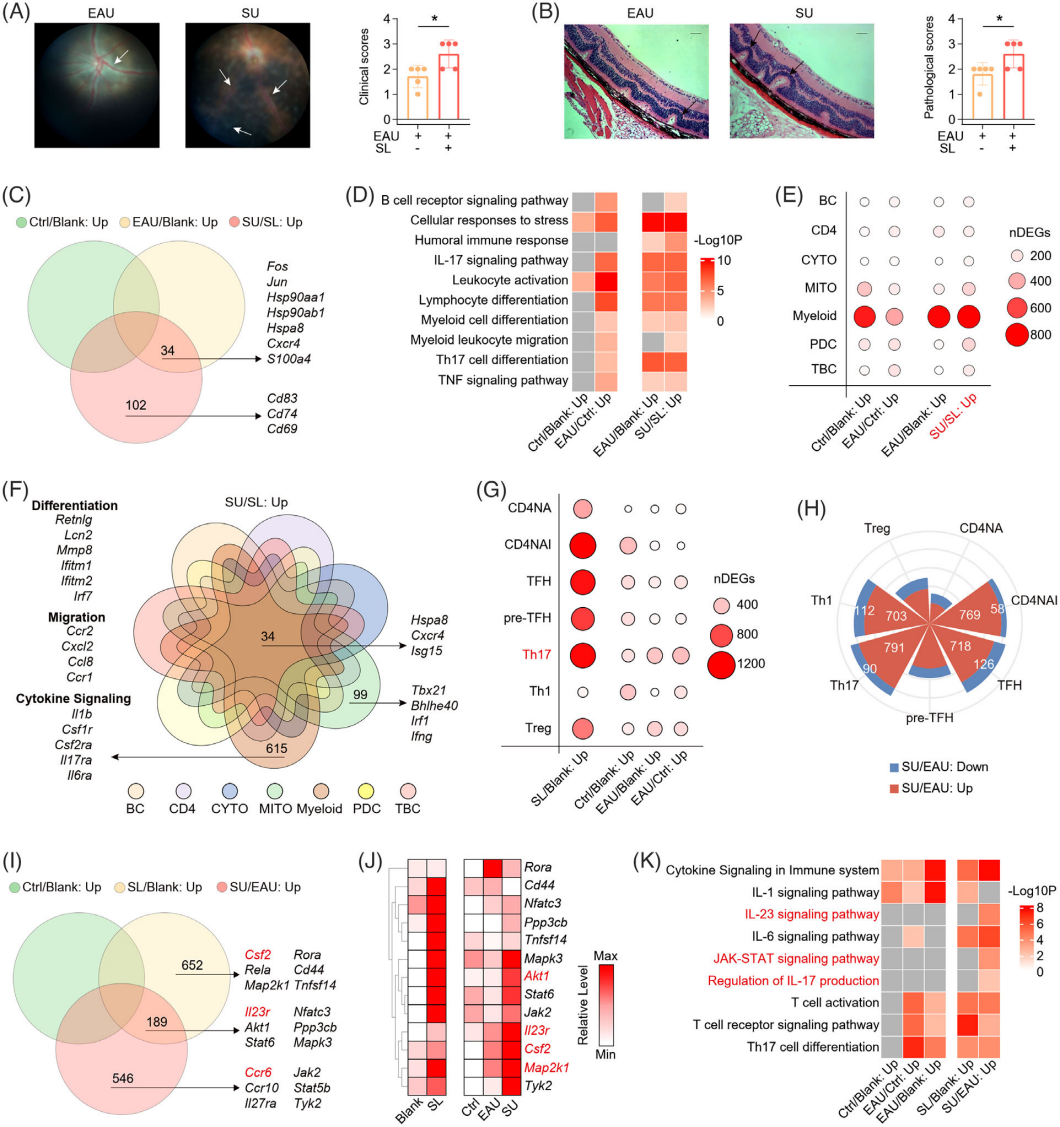
SL promoted EAU development and enhanced the pathological immune response to EAU challenge. (A) The representative fundus images after immunization at day 14 (left). The white arrows indicate inflammatory exudation and linear lesions. The column charts showing the clinical scores between two groups (n = 5/group, right). Data represented as mean ± SD. Significance was determined using two‐tailed unpaired student's *t*‐test; *p < .05. (B) The representative H&E‐stained images after immunization at day 14 (left). Scale bars: 50 μm. The black arrows indicate retinal folding. The column charts showing the histological scores between two groups (n = 5/group, right). Data represented as mean ± SD. Significance was determined using two‐tailed unpaired student's *t*‐test; *p < .05. (C) Venn diagram showing the interactions of upregulated DEGs in Ctrl/Blank, EAU/Blank and SU/SL comparisons. (D) Representative GO biological process and pathways enriched in upregulated DEGs in Ctrl/Blank, EAU/Ctrl, EAU/Blank and SU/SL comparisons. The grey indicates inexistence in this group. (E) The dot plot showing the number of upregulated DEGs in Ctrl/Blank, EAU/Ctrl, EAU/Blank and SU/SL comparisons. (F) Venn diagram showing the interactions of upregulated DEGs in SU/SL comparison among immune sub‐populations. (G) The dot plot showing the number of upregulated DEGs in SL/Blank, Ctrl/Blank, EAU/Blank and EAU/Ctrl comparisons in CD4^+^ TC subsets. (H) The rose diagram showing the number of DEGs in SU/EAU comparison among CD4^+^ TC subsets. (I) Venn diagram showing the interactions of upregulated DEGs in Ctrl/Blank, SL/Blank and SU/EAU comparisons in Th17 cells. The genes listed are those related with Th17‐cells activation. (J) The heatmap showing the relative levels of genes obtained from Figure 3I in Th17 cells among five groups. Colour scheme is based on *z*‐score distribution from minimum (white) to maximum (red). (K) Representative GO biological process and pathways enriched in upregulated DEGs of Th17 cells in Ctrl/Blank, EAU/Ctrl, EAU/Blank, SL/Blank and SU/EAU comparisons. The grey indicates inexistence in this group.

SL has been linked to increased autoimmune tendencies.^36^, ^37^ An existing clinical study has shown increased recurrence of AU induced by SL. To investigate how SL can increase AU recurrence, we explored how SL influences EAU development and related autoimmune responses.^13^ We evaluated symptoms and graded the clinical scores in different groups of mice by photographing the fundus. In EAU mice immunized with adjuvants plus IRBP_1−20_, manifestation in the fundus included multiple chorioretinal lesions and infiltrations with retinal detachment (Figure 3A). These EAU symptoms were not presented in the blank, SL and control group (Supporting Information Figure S4A). Haematoxylin and eosin‐stained eyeball sections exhibited cell infiltration and retinal folding with detachment (Figure 3B). Intriguingly, SU mice exhibited aggravated EAU symptoms and worse retinal folding compared to EAU mice (Figure 3A,B). This divergence in EAU severity corresponded with clinical findings that indicated SL aggravated AU.

To capture the unique gene signature of IRBP_1−20_‐immunized EAU mice, we conducted DEGs analysis between the control group and the blank group and between the EAU and control group. In contrast to the blank mice, control group showed increased level of inflammation‐related genes (*S100a8* and *S100a9*), interferon‐related genes (*Ifitm3*, *Irf7*), and genes related to negative regulation of immune response (*Socs1* and *Nfkbia*) (Supporting Information Figure S4B). In contrast, compared to control mice, EAU group upregulated the genes related to lymphocyte activation (*Cd69, Cxcr4*), Th17 activation (*Pim1*, *Fos*, *Jun*, *Jund* and *Hsp90aa1*) and humoral immunity (*Ighg1*, *Mzb1* and *Jchain*) (Supporting Information Figure S4C). Functional analysis of these DEGs demonstrated that control group increased pathways related to cell activation and immune response, and these processes were further enriched in EAU group. Notably, IL‐17, MAPK signalling pathway and BC‐mediated immunity were uniquely increased in EAU groups (Supporting Information Figure S4D). These EAU‐specific pathways and processes were closely related to Th17 activation, antibodies production and autoimmune response. Collectively, although the injection of Freund's adjuvant and PTX might result in general inflammation, autoimmune‐related pathways were specifically enriched in IRBP_1−20_‐immunized EAU mice.

To explore the effects of SL on the transcription level in response to EAU, we identified the upregulated DEGs between SU and SL mice, and the upregulated DEGs in EAU/blank comparison. We also removed the upregulated DEGs between control and blank group to investigate genes exclusively upregulated in EAU groups (Figure 3C). The common DEGs exclusively upregulated in EAU groups included the AP‐1, heat shock protein families, *Cxcr4* and *S100a4*. Notably, we also identified SL‐specific genes during EAU including activation gene *Cd69* and the inflammatory genes *Cd83 and Cd74* (Figure 3C). We next performed a functional analysis for each comparison (Figure 3D) and found that EAU induced the increase of Th17‐cell differentiation, humoral immune response and myeloid cell differentiation, which were more pronounced after SL. Additionally, several processes were exclusively enriched in SU/SL comparison including BC receptor signalling pathway and myeloid leukocyte migration (Figure 3D). These results indicate SL enhances the inflammatory and autoimmune responses during EAU challenge.

We also explored the impact of SL on each sub‐population. The number of DEGs in response to EAU was increased after SL (Figure 3E). Next, we generated a Venn diagram to explore the cell‐specific upregulated DEGs in the SU/SL comparison, and we found that autoimmune‐related genes, including *Hspa8*, *Cxcr4* and *Isg15*, were upregulated in all immune cell subsets (Figure 3F). T‐cell differentiation‐related genes (*Tbx21*, *Bhlhe40*, *Irf1* and *Ifng*) were upregulated in MITO cells. Notably, in myeloid cells, the expression of differentiation‐, migration‐ (*Ccr2*) and cytokine signalling‐related genes (*Il1b*, GM‐CSF receptors *Csf1r*, *Csf2ra*, IL‐17 receptor *Il17ra* and IL‐6 receptor *Il6ra*) increased in SU group compared to SL group (Figure 3F). Collectively, SL upregulated several cell‐specific pathological immune responses during EAU challenge like myeloid cell activation.

Next, we explored the influence of SL on CD4^+^ subsets in EAU mice. Increased DEGs were detected in the SL/blank compared to the EAU/blank and control/blank comparisons, indicating that SL affects CD4^+^ subsets more broadly than EAU (Figure 3G). In particular, Th17 cells were most affected by EAU across CD4^+^ subsets based on the count of upregulated DEGs in both EAU/blank and EAU/control comparisons (Figure 3G). When exploring the effects of SL on EAU based on SU/EAU comparison, we found that the number of upregulated DEGs was higher than that of downregulated DEGs, which potentially contributed to the aggravated EAU symptoms. Moreover, SU mice affected Th17 cells more than in any other CD4^+^ subsets based on the DEGs numbers in SU/EAU comparison (Figure 3H).

To identify SL‐induced changes in Th17 cells during EAU, we analysed the upregulated DEGs from the SL/blank and SU/EAU comparisons, and removed the upregulated DEGs in control/blank comparison. We identified several genes that are involved in Th17 cells activation (Figure 3I). SL increased level of *Il23r* and *Csf2* (encoding GM‐CSF), both related to Th17 differentiation and pathogenicity. IL‐23 can act on IL‐23R on CD4^+^ TCs to facilitate Th17 differentiation and GM‐CSF secretion, indicating that GM‐CSF and IL‐23R were the hallmarks of pathogenic Th17 cells.^38^ In addition, SU increased the expression of *Ccr6, Jak2* compared to EAU mice (Figure 3I). Further analysis indicated that several genes were not increased in control mice, but increased during EAU induction and enhanced by SL like *Csf2*, *Il23r*, *Akt1* and *Map2k1* (Figure 3J). Thus, we conducted the functional analysis of upregulated DEGs in different comparisons (Figure 3K). The upregulated DEGs in EAU/blank and EAU/control comparisons were enriched in Th17‐cell differentiation, T‐cell activation and T‐cell receptor signalling pathway, while these processes were not presented in control/blank comparison. In addition, several processes were increased in SU/EAU comparison like IL‐17 production and JAK‐STAT signalling pathway. Moreover, the IL‐23 signalling pathway was uniquely elevated in SU mice compared to EAU mice, yet with no alteration in the IL‐1 signalling pathway (Figure 3K). The heterogeneity of these Th17 differentiation‐related processes highlights the role of SL in promoting pathological immune responses and the IL‐23 signalling pathway during EAU.

In summary, SL aggravated EAU symptoms and pathological immune responses, especially the differentiation and activation of Th17 cells.

### 2.5 SL results in aberrant intercellular interaction patterns during EAU

**FIGURE 4:**
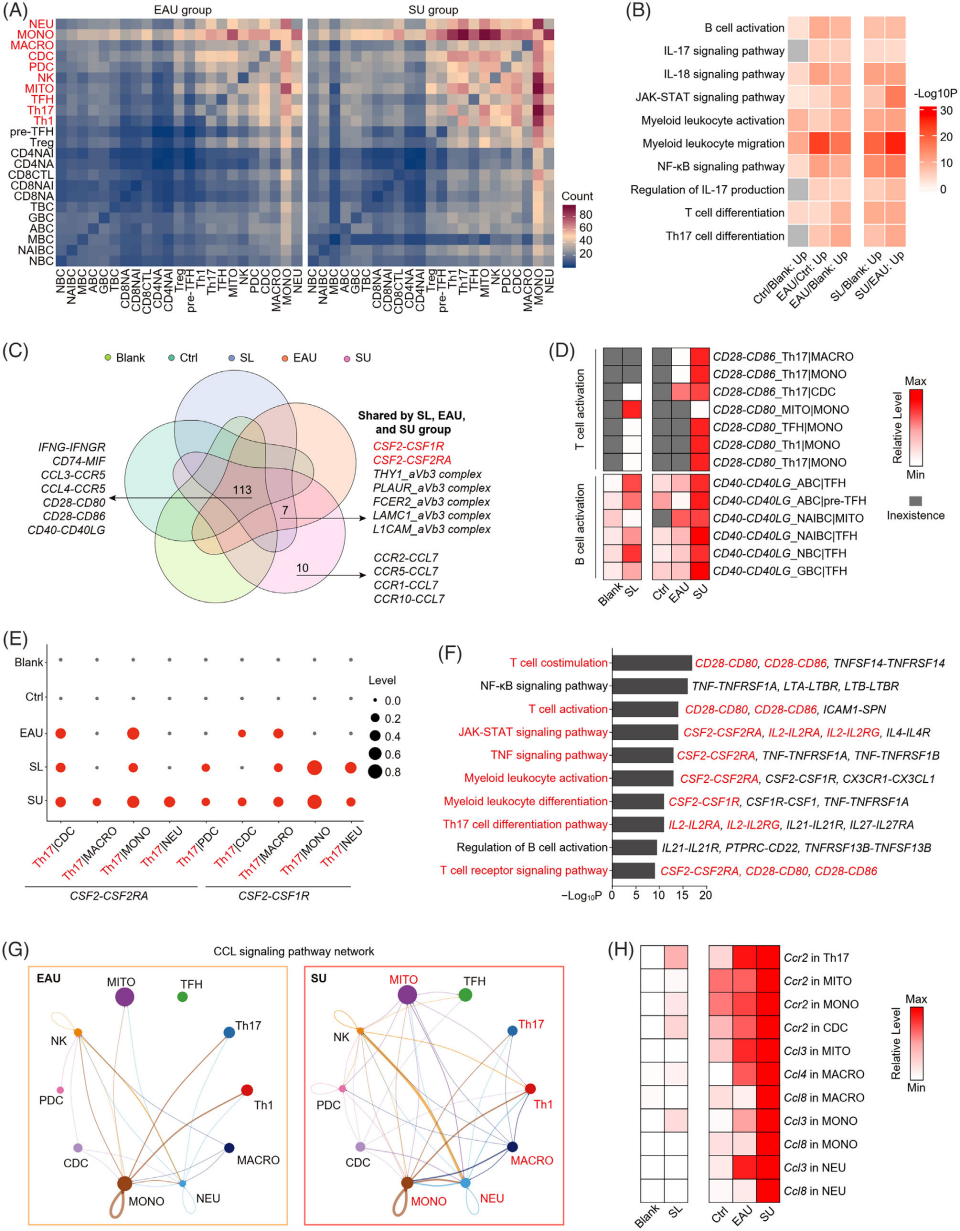
SL resulted in aberrant intercellular interaction patterns during EAU. (A) The heatmap showing the number of the predicted cell–cell interaction between EAU and SU groups. CellPhoneDB was used to calculate and predict the possible cell–cell interaction. (B) Representative GO biological process and pathways enriched in upregulated cell–cell interaction in Ctrl/Blank, EAU/Ctrl, EAU/Blank, SL/Blank and SU/EAU comparisons. The grey indicates inexistence in this group. (C) Venn diagram showing the interactions of predicted cell–cell interaction among five groups. (D) The heatmap showing the relative levels of interaction related to T‐ and B‐cells activation among five groups. Colour scheme is based on *z*‐score distribution from minimum (white) to maximum (red). The grey indicates inexistence in this group. (E) The dot plot showing the relative levels of GM‐CSF‐related interaction among five groups. The means of the expression level of *CSF2* in Th17 and its receptors *CSF2RA*, *CSF1R* in myeloid subsets are scaled. (F) Representative GO biological process and pathways enriched in upregulated cell–cell interaction in SU/EAU comparison. (G) Circle plot showing CCL signalling pathway network between EAU and SU groups. (H) The heatmap showing the levels of ligands and receptors related to CCL signalling in immune cells among five groups. Colour scheme is based on *z*‐score distribution from minimum (white) to maximum (red).

Cell‐cell interactions facilitate cell functions and immune homeostasis.^39^, ^40^ Aberrant intercellular communication is associated with various inflammatory and autoimmune disorders.^41^, ^42^ To identify the impact of SL and EAU on cellular communication, we performed bioinformatics analyses of intercellular interactions by referring to the level of ligand‐receptor pairs in immune cells using CellPhoneDB.^43^ We compared the number of CellPhoneDB‐predicted interactions and observed that both EAU and SL increased immune cell interactions, especially among the Th1, Th17 and myeloid subsets (Figure 4A, Supporting Information Figure S5A). To explore the functions of ligand‐receptor interactions during SL and EAU, we conducted functional enrichment analysis of the upregulated ligand‐receptor pairs in the control/blank, EAU/control, EAU/blank, SL/blank and SU/EAU comparisons. Consistent with the aforementioned proinflammatory effects of SL, the upregulated ligand‐receptor pairs in SL/blank comparison were enriched in several autoimmune and inflammatory processes like myeloid leukocyte migration, BC activation, NF‐κB and JAK‐STAT signalling pathways. Notably, SU mice enhanced those increased processes in IRBP_1−20_‐immunized EAU, including IL‐17 production, IL‐17 signalling pathway and Th17‐cell differentiation (Figure 4B).

We further performed the integrated comparative analysis of the predicted interactions among the five groups and identified 10 ligand‐receptor pairs uniquely upregulated in SU mice including the interaction of CCL7 and its receptors in chemokine pathways (Figure 4C). Among these 113 common pairs shared by all groups, IFN‐γ and its receptors have been demonstrated to correlate with autoimmunity.^44^ *CD74*‐*MIF* and *CCL3*/*4*‐*CCR5* interacting pairs function in lymphocyte activation and migration.^45^, ^46^ Moreover, *CD28*‐*CD80*/*CD86* and *CD40*‐*CD40LG* interactions are associated with the TCs and BCs activation, respectively.^47^, ^48^ Among the ligand‐receptor pairs associated with T‐cell activation, the *CD28*‐*CD86* interaction pair among Th17 and myeloid cells was specifically presented after SL, simultaneously with the SL‐induced upregulation of *CD28*‐*CD80* interaction pair among TC subsets and myeloid cells. In BCs and TCs, *CD40*‐*CD40LG* interactions related to B‐cell activation were enhanced by SL and EAU (Figure 4D). In addition, ligand‐receptor pairs in the IFN‐γ and MIF pathways were upregulated with SL and EAU (Supporting Information Figure S5B). Next, we analysed the shared ligand‐receptor pairs between SL and EAU to explore the underlying mechanisms of SL on EAU exacerbation. Notably, among these shared pairs in SL, EAU and SU mice, GM‐CSF and its receptors are known as the key mediators in autoimmunity.^49^ We discovered that GM‐CSF interacted with its receptors mainly in Th17 and myeloid cell subsets, which was upregulated after SL (Figure 4C,E). We also found in SU mice that GM‐CSF and its receptors contributed to the JAK‐STAT and TNF signalling pathways, myeloid leukocyte differentiation and activation, all of which exacerbating EAU symptoms (Figure 4F). These results indicate that SL induces the development of EAU by promoting the binding of GM‐CSF and corresponding receptors between Th17 and myeloid cells.

We then explored intercellular signalling using CellChat, a tool to analyse cell‐cell networks and predict major inputs and outputs of signalling pathways.^43^ The analysis suggested that compared with EAU mice, SU mice had increased CCL signalling pathway function in TC subsets (MITO, Th17 and Th1) and myeloid cells (monocytes, macrophages and neutrophils) (Figure 4G). By exploring the levels of genes related to CCL signalling, we found that SU mice upregulated *Ccr2* in Th17, MITO, monocyte and CDC and chemokine ligands in myeloid cell subsets (Figure 4H). Additionally, the IL‐2 signalling pathway, which regulates T‐cell proliferation and activation,^50^ was detected in SL and SU but not in blank, control and EAU mice (Supporting Information Figure S5C), suggesting a potential connection between SL and IL‐2 signalling activation. TC subsets (MITO, TFH, Th17 and Th1) are the major sources of IL‐2, which works in an autocrine or paracrine manner.

Collectively, we identified the aberrant cell‐cell interaction patterns associated with increased T‐cell activation, Th17 pathogenicity, myeloid cells activation and migration. The binding of GM‐CSF and its receptors between Th17 cells and myeloid cells might result in the aggravated EAU after SL.

### 2.6 Anti‐GM‐CSF treatment rescued the SL‐induced infiltration of pathogenic Th17 and myeloid cells

**FIGURE 5:**
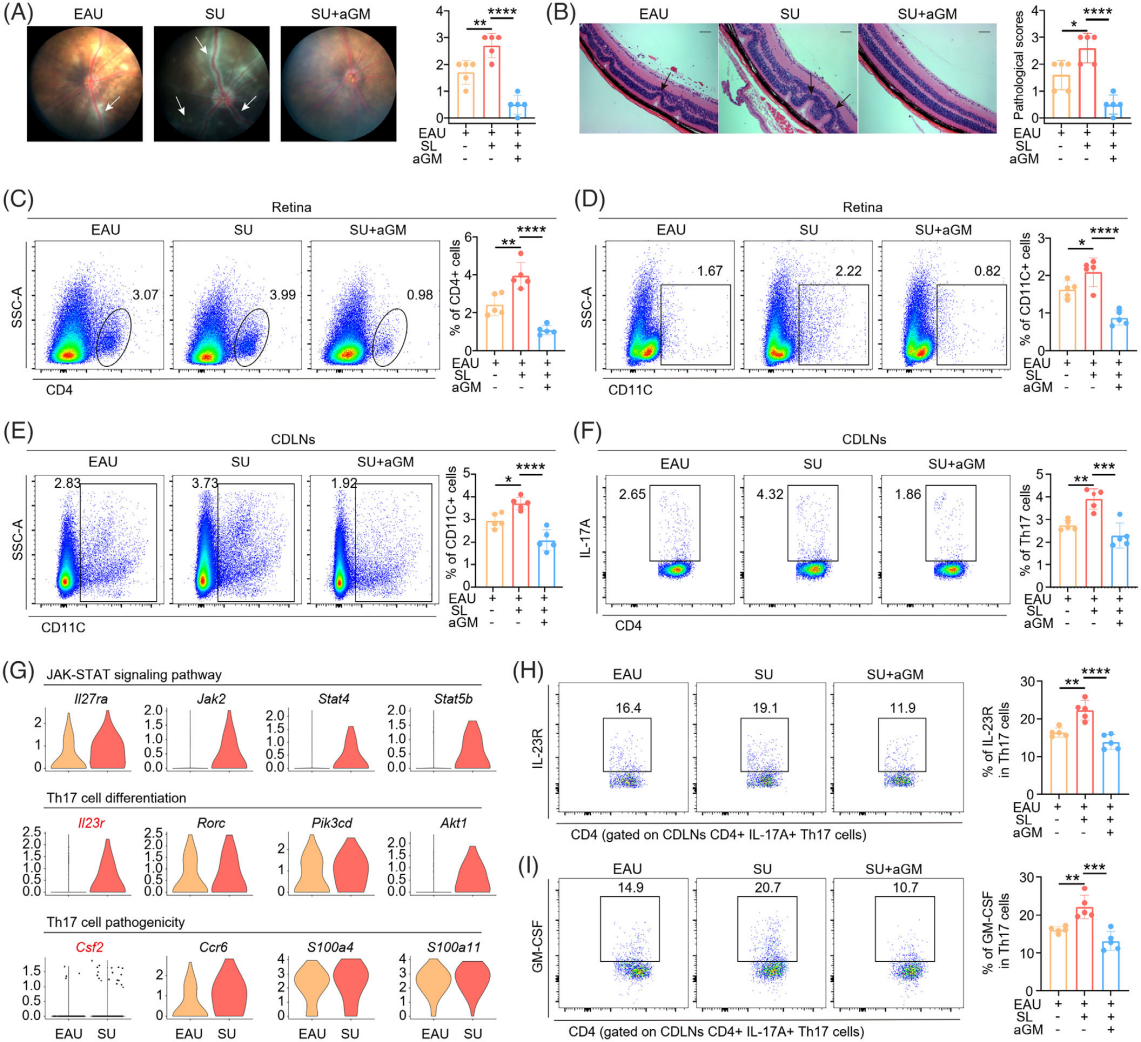
Anti‐GM‐CSF treatment displayed rescue effects for the SL‐induced aggravation of EAU. (A) The representative fundus images after immunization at day 14 (left). The white arrows indicate inflammatory exudation and linear lesions. The column charts showing the clinical scores among three groups (n = 5/group, right). Data represented as mean ± SD. Significance was determined using one‐way ANOVA; **p < .01, ****p < .0001. (B) The representative H&E‐stained images after immunization at day 14 (left). Scale bars: 50 μm. The black arrows indicate retinal folding. The column charts showing the histological scores among three groups (n = 5/group, right). Data represented as mean ± SD. Significance was determined using one‐way ANOVA; *p < .05, ****p < .0001. (C‐D) Retinal cells from EAU, SU and SU + aGM mice were obtained after immunization at day 14. Flow cytometry showed the proportion of CD4^+^ TC (**C**) and CD11C^+^ cells (**D**) (n = 5/group). Data represented as mean ± SD. Significance was determined using one‐way ANOVA; *p < .05, **p < .01. and ****p < .0001. (E‐F) CDLNs cells from EAU, SU and SU + aGM mice were obtained after immunization at day 14. Flow cytometry showed the proportion of CD11C^+^ cells (**E**) and Th17 cells (**F**) (n = 5/group). Data represented as mean ± SD. Significance was determined using one‐way ANOVA; *p < .05, **p < .01, ***p < .001 and ****p < .0001. (G) The violin plot showing the level of genes related to JAK‐STAT signalling pathway, Th17‐cell differentiation pathway and Th17‐cell pathogenicity pathway in Th17 cells between EAU and SU groups. (H‐I) CDLNs cells from EAU, SU and SU + aGM mice were obtained after immunization at day 14. Flow cytometry showed the proportion of IL‐23R^+^ cells (**H**) and GM‐CSF^+^ cells (**I**) in gated CD4^+^IL17A^+^ Th17 cells (n = 5/group). Data represented as mean ± SD. Significance was determined using one‐way ANOVA; **p < .01, ***p < .001 and ****p < .0001.

Our data above demonstrated that SL aggravated EAU symptoms and increased relevant pathological immune responses, especially the production of GM‐CSF by Th17 and the intercellular communication between Th17 and myeloid cells. Thus, we explored whether inhibition of GM‐CSF can ameliorate SL‐induced EAU development. We first evaluated EAU severity in EAU, SU and SU mice treated with anti‐GM‐CSF antibody. In contrast to EAU mice, the SU mice exhibited more severe EAU manifestations, characterised by higher clinical and pathological scores, which were alleviated by anti‐GM‐CSF treatment (Figure 5A,B). Our findings indicated the efficacy of anti‐GM‐CSF therapy for alleviating SL‐induced AU recurrence.

Moreover, since eye infiltration by inflammatory cells is a crucial pathologic progression of EAU,^51^, ^52^ we conducted flow cytometry analysis to identify any change in CD4^+^ TCs in EAU and SU mice. Increased CD4^+^ TCs were detected in the eyes of SU mice compared with EAU mice. After anti‐GM‐CSF treatment, this infiltration was significantly reduced (Figure 5C, Supporting Information Figure S6A). In addition, CD11C^+^ cells are crucial myeloid cells that preserve antigen presenting function, serving as principal drivers of autoimmune‐related pathology and have been used in studies of the mutual effect between Th17 cells and antigen presenting cells.^38^, ^53^, ^54^ Additionally, the level of infiltrated CD11C^+^ myeloid cells under the anti‐GM‐CSF treatment displayed trends consistent with those of CD4^+^ TCs (Figure 5D). These findings indicate that anti‐GM‐CSF treatment decreased intraocular inflammation, which is consistent with the clinical and histopathological results.

We also collected and analysed immune cells from CDLNs to identify whether SL induced abnormal immune system function during EAU development.^55^ Using flow cytometry, we detected increased CD11C^+^ and Th17 cells in CDLNs of SU mice compared to EAU mice, indicating elevated infiltration of autoimmune‐related myeloid and pathological Th17 cells (Figure 5E,F & Supporting Information Figure S6B,C). Anti‐GM‐CSF antibody reduced the proportion of these cell types in the CDLNs of SU mice. In addition, we also detected the level of serum immunoglobulins G (IgG) specific to IRBP_1−20_ (anti‐IRBP_1−20_ IgG) using enzyme‐linked immunosorbent assay (ELISA). The serum anti‐IRBP_1−20_ IgG were increased in SU group compared to EAU mice, and these upregulations were inhibited by anti‐GM‐CSF treatment (Supporting Information Figure S6D).

**FIGURE 6:**
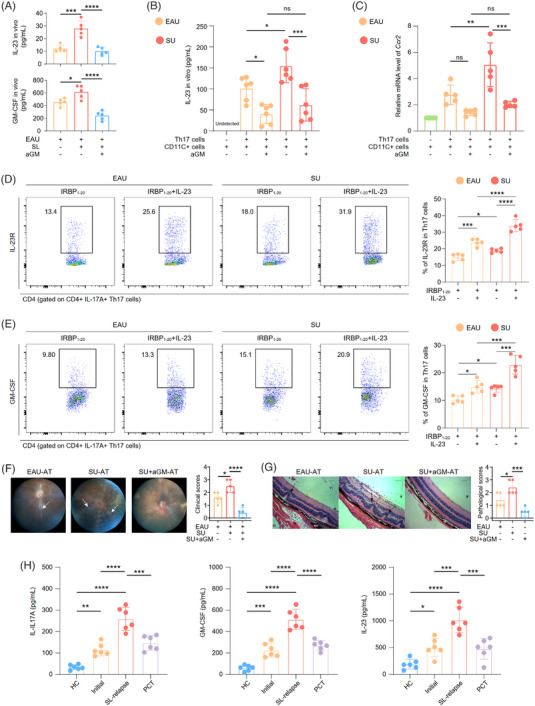
SL increased Th17 pathogenicity by enhancing the IL‐23–Th17–GM‐CSF positive feedback mechanism. (A) Serum from EAU, SU and SU + aGM mice were obtained after immunization at day 14. The column charts showing the serum level of IL‐23 and GM‐CSF among three groups (n = 5/group). Data represented as mean ± SD. Significance was determined using one‐way ANOVA; *p < .05, ***p < .001 and ****p < .0001. (B‐C) After 48‐h co‐culture of Th17 with CD11C^+^ cells with or without anti‐GM‐CSF antibody, the supernatant level of IL‐23 was measured by ELISA (**B**), and the mRNA level of Ccr2 was detected by real‐time quantitative PCR (**C**). Data represented as mean ± SD. Significance was determined using one‐way ANOVA; ns, not significant, *p < .05, **p < .01 and ***p < .001. (D‐E) CD4^+^ T cells from EAU and SU groups were cultured with IRBP_1‐20_ alone or with IRBP_1‐20_ plus with IL‐23 for 72 h. The flow cytometry histograms (left) and column charts (right) showing the percentage of IL‐23R^+^ (**D**) and GM‐CSF^+^ cells (**E**) in CD4^+^IL‐17A^+^ Th17 cells (n = 5/group). Data represented as mean ± SD. Significance was determined using one‐way ANOVA; *p < .05, ***p < .001, ****p < .0001. (F) The representative fundus images after adoptive induction by pathological TCs from EAU (EAU‐AT), SU (SU‐AT) or SU plus anti‐GM‐CSF treatment (SU + aGM‐AT) groups (left). The white arrows indicate inflammatory exudation and linear lesions. The column charts (right) showing the clinical scores among three groups (n = 5/group). Data represented as mean ± SD. Significance was determined using one‐way ANOVA; *p < .05, ****p < .0001. (G) The representative H&E‐stained images after adoptive induction by pathological TCs from EAU (EAU‐AT), SU (SU‐AT) or SU plus anti‐GM‐CSF treatment (SU + aGM‐AT) groups (left). Scale bars: 50 μm. The black arrows indicate retinal folding. The column charts (right) showing the histological scores among three groups (n = 5/group). Data represented as mean ± SD. Significance was determined using one‐way ANOVA; *p < .05, ***p < .001. (H) The column charts showing the supernatant level of IL‐17A, GM‐CSF and IL‐23 among HC and uveitis patients under different phases (n = 6/group). Data represented as mean ± SD. Significance was determined using one‐way ANOVA; *p < .05, **p < .01, ***p < .001, ****p < .0001.

Th17 cells play key roles in the onset and aggravation of EAU.^56^ Compared to EAU mice, we found that SU mice not only increased Th17 frequency, but also upregulated genes associated with JAK‐STAT signalling pathway, Th17‐cell differentiation and Th17‐cell pathogenicity, like *Csf2* (encoding GM‐CSF) and *Il23r* in Th17 cells (Figure 5G). Thus, we validated the GM‐CSF and IL‐23R levels in Th17 cells in vivo. We found elevated levels of both proteins in SU mice compared to EAU mice, which were inhibited with anti‐GM‐CSF treatment (Figure 6H,I & Supporting Information Figure S6E).

In conclusion, these results showed that SL induces the infiltration of CD11C^+^ myeloid and pathogenic Th17 cells both in the eyes and CDLNs, thus, promoting EAU development. Anti‐GM‐CSF treatment significantly rescued SL‐induced inflammatory activation in EAU.

### 2.7 SL increased Th17 pathogenicity by enhancing the IL‐23–Th17–GM‐CSF positive feedback mechanism

After SL, EAU mice exhibited upregulated GM‐CSF levels in Th17 cells (Figure 3J) and intercellular communication of GM‐CSF and its receptors on Th17 and myeloid cells (Figure 4E). GM‐CSF, secreted by Th17 cells, acts on the GM‐CSF receptors on myeloid cells and induces myeloid cells activation and migration. IL‐23 secreted by myeloid cells may in turn bind to IL‐23R on Th17 cells and promoted Th17 pathogenicity, therefore, constructing a positive feedback mechanism to maintain myeloid cells activation and Th17 pathogenicity.^38^ Considering our data of scRNA‐seq that GM‐CSF and IL‐23R levels in Th17 cells (Figure 3J), as well as GM‐CSF receptor level in myeloid cells (Figure 3F) were increased in SU mice, we hypothesised that SL may promote the pathogenesis of EAU through enhancing the IL‐23–Th17–GM‐CSF positive feedback loop. Indeed, we detected increased concentration of GM‐CSF and IL‐23 in serum from SU mice, which then decreased by anti‐GM‐CSF treatment (Figure 6A).

To investigate this hypothesis, we isolated and co‐cultured CD11C^+^ and CD4^+^CCR6^+^CXCR3^−^ Th17 cells from EAU and SU mice. The anti‐GM‐CSF antibody or recombinant GM‐CSF were also added. IL‐23 secretion from CD11C^+^ cells was enhanced by co‐culturing with Th17 cells, with SU‐derived cells exhibiting the highest levels of secretion (Figure 6B). Moreover, anti‐GM‐CSF treatment decreased IL‐23 secretion (Figure 6B), and addition of GM‐CSF could increase IL‐23 level in the supernatant (Supporting Information Figure S6F), suggesting that GM‐CSF from Th17 cells served as an inducer for IL‐23 production from CD11C^+^ cells. Studies have reported that GM‐CSF could promote the activation and migration of monocytes.^57^ In addition, we found that co‐culturing with Th17 cells also enhanced the expression of *Ccr2*, which was promoted in SU‐derived cells and then inhibited by anti‐GM‐CSF treatment (Figure 6C). These results suggested that pathological GM‐CSF derived from Th17 cells could augment the activation and migration of myeloid cells, which were line with the infiltration of CD11C^+^ cells in the eyes and CDLNs, resulting in the SL‐induced EAU aggravation.

The above findings demonstrated that Th17‐derived GM‐CSF participated in inducing IL‐23 secretion in myeloid cells. Moreover, high level of GM‐CSF from Th17 cells together with consequently high IL‐23 from myeloid cells may further enhance Th17 pathogenicity through the strengthened IL23‐IL‐23R interaction pair. To confirm this feedback mechanism, we used IRBP_1−20_ as a stimulator. CDLN‐derived lymphocytes from EAU and SU mice were stimulated with IRBP_1−20_ or IRBP_1−20_ plus IL‐23. IL‐23 increased IL‐23R and GM‐CSF in Th17 cells with a greater degree in SU mice in contrast to that in EAU mice (Figure 6D,E), which was consistent with the scRNA‐seq results (Figure 3J). In addition, anti‐IL‐23 antibody decreased Th17 frequency and GM‐CSF production of Th17 cells (Supporting Information Figure S7A,B). These findings confirmed that SL strengthens GM‐CSF production and Th17 pathogenicity by upregulating IL‐23/IL‐23R signalling pathways. We also performed an adoptive transfer experiment. The SU‐derived lymphocytes induced more severe uveitis in EAU mice (Figure 6F,G). After treatment with anti‐GM‐CSF, SU‐derived lymphocytes failed to induce uveitis (Figure 6F,G). Collectively, these results demonstrated that SL enhanced Th17 pathogenicity and EAU exacerbation by promoting the IL‐23–Th17–GM‐CSF positive feedback loop.

Additionally, we explored whether the SL‐induced relapse of AU was associated with the IL‐23–Th17–GM‐CSF feedback loop clinically. In addition to the blood samples from healthy individuals with or without SL, we also collected blood samples from AU patients in initial stage and SL‐induced recurrent AU (Supporting Information Table S2). Patients with SL‐induced recurrent uveitis are defined as uveitis patients who displayed stable disease condition for more than 3 months, but appeared to have obvious relapsing symptoms including redness and pain in the eye, vision loss, anterior chamber flash and cell rating increased by two grades with or without macular oedema after 1 to 3 days of working through the night, despite excluding factors such as diet, medication and emotion. As for patients with SL‐induced recurrent uveitis, we have treated them with conventional therapy. After treatment, the patients recuperated to stability for at least 1 month, characterised by restored vision and resumed the prescription of steroids at maintenance dosage. Under these circumstances, peripheral blood from these patients was collected for the detection of inflammatory cytokines. Flow cytometry indicated that SL increased the level of GM‐CSF in Th17 cells and IL‐23 in CD14^+^ monocytes both in PBMCs of healthy individuals and AU patients (Supporting Information Figure S7C‐F). These cytokines might contribute to the enhanced interaction between Th17 cells and myeloid cells and account for the SL‐induced AU relapse. Indeed, serum from patients in the initial phase showed increased levels of IL‐17A, GM‐CSF and IL‐23 compared to healthy individuals. Notably, these upregulations were enhanced in patients with SL‐induced recurrent AU (Figure 6H), which was consistent with our findings in EAU mice. These inflammatory cytokines were also downregulated after conventional therapy. These results indicated that it contributed to the SL‐induced relapse of AU. This suggests that the clinical recurrence of AU caused by SL may be attributed to the enhanced IL‐23–Th17–GM‐CSF positive feedback mechanism and increased inflammatory cytokines (Supporting Information Figure S8).

## 3 DISCUSSION

Here, we describe an SL‐specific immune cell atlas derived from human blood samples and mouse CDLNs at the single‐cell level. By combining CyTOF and scRNA‐seq, we evaluated SL‐induced alterations in immune cell percentage, subset‐specific gene expression, dominant pathways and intercellular interactions. Furthermore, we demonstrated the effects of SL on EAU development and EAU‐specific immune responses. The major results are: (1) SL‐induced inflammation in humans by remodelling the cells composition and elevating autoimmune‐related cytokines in effector CD4^+^ TCs and myeloid cells; (2) immune cells in mice CDLNs after SL had enhanced inflammation, increased autoimmune‐associated genes and enriched signalling pathways in TC, BC and myeloid subsets; (3) SL exacerbated EAU manifestation and increased the pathogenic Th17 and CD11C^+^ cells in EAU mice; (4) the SL‐induced EAU aggravation can be attributed to the upregulation of genes and pathways involved in autoimmune responses, especially the differentiation and pathogenicity of Th17 cells, as well as the activation and migration of myeloid cells; (5) SL induced the upregulation of specific cell‐cell interactions among TCs and myeloid subsets during EAU; (6) SL enhanced Th17 pathogenicity and myeloid cells activation through the IL‐23–Th17–GM‐CSF feedback loop, thus, promoting EAU development; (7) anti‐GM‐CSF treatment reduced the effects of SL on Th17 pathogenicity and EAU exacerbation and (8) SL‐induced relapse of human AU was associated with the IL‐23–Th17–GM‐CSF feedback loop clinically.

SL has become commonplace among the labour force.^2^ SL‐induced dysregulated immune states, accompanied by increased risk of cardiovascular and metabolic diseases, have been associated with higher adverse health outcomes and mortality risk.^5^, ^58^ Sleep deprivation promotes experimental pulmonary metastasis by altering the tumour immune response in mice^59^; however, how SL affects the immune system and increases autoimmunity was unknown. Here, we combined high‐throughput CyTOF and scRNA‐seq to construct an immune atlas detailing how SL influenced the immune cells. In mice, SL altered proportions of immune cells and upregulated inflammatory pathways (IL‐6, JAK‐STAT, MAPK and NF‐κB), and stress response. Similar results were found in human blood samples in our CyTOF data, as shown by the increased CMC frequency and elevated inflammatory cytokine production including IL‐1β, GM‐CSF and TNF‐α. CMC is the major component of monocytes that promote inflammation and phagocytosis.^60^ This monocyte lineage is involved in SL‐mediated altered cerebral reactions.^61^ SL was previously proposed to initiate inflammatory cytokine storms and exacerbate inflammatory disorders including COVID‐19 infections.^62^ Furthermore, we discovered that SL induces an autoimmune predisposition. SL primarily affects Th17 cells, indicated by increased differentiation‐ and activation‐related genes (*Il23r*, *Rorc* and *Csf2*). SL also affected TFH, a CD4^+^ subset involved in TC and BC interaction, by upregulating *Cxcr5* and *Ccr6*. As CXCR5^+^CCR6^+^ TFH cells may participate in the antibody‐associated immune responses in Sjögren's syndrome,^63^ our results indicate the involvement of this TFH subset in SL‐induced immune dysregulation. Finally, our results suggest SL enhances intercellular communication, especially between myeloid cells and pathogenic CD4^+^ subsets, providing another mechanism underlying how SL induces autoimmune predisposition. Generally, SL induces abnormal differentiation and activation of immune cells in humans and mice.

SL causes alterations in immune populations that can lead to autoimmunity. Sleep deprivation induces an earlier onset of SLE in predisposed mice.^36^ Even so, few studies have investigated how SL affects autoimmunity. In this study, we combined a mouse model and scRNA‐seq to demonstrate the effects and underlying mechanisms of SL on EAU. We detected exacerbated EAU symptoms in SU mice, accompanied by the activation and infiltration of CD11C^+^ and CD4^+^ cells in mouse retina and CDLNs. Next, we noted the cellular and molecular alterations using single‐cell technologies. Following SL, autoimmune‐related genes (S100 family) and pathways were upregulated in CDLNs immune cells. Overexpression of S100 family genes, which are biomarkers for RA and IBD, can stimulate leukocyte recruitment and cytokine secretion, thus, initiating multiple autoimmune disorders.^64^ The combined effect of S100A8, TNF‐α, IL‐1β and IL‐17 induced activated matrix metalloproteinases and exacerbated arthritis.^65^ In addition, we introduced control group into our study design to eliminate the influence of adjuvants during the establishment of EAU mice model. Data show that adjuvants, including Freund's adjuvant and PTX, increased general inflammatory status. However, the IL‐17 signalling pathway and Th17 cell differentiation, which were uniquely upregulated in IRBP_1−20_‐immunized EAU and promoted by SL, represent the specific effects of SL on EAU‐specific autoimmune response. We next focused on Th17 cells and demonstrated that both EAU and SL primarily influenced Th17 and enhanced Th17‐cell activation. For example, SL increased EAU‐induced Th17 infiltration and upregulated inflammation‐ and autoimmune‐related pathways (IL‐17, IL‐23 and JAK‐STAT). As downstream effectors of pro‐inflammatory cytokines, the JAK‐STAT pathway is involved in inflammatory processes. Clinical trials are on‐going for JAK inhibitors as autoimmune treatments including AU.^27^, ^66^ More importantly, we discovered that SL enhanced inter‐cellular communication among myeloid cells, TCs, and BCs, contributing to the activation and differentiation of TCs (CD28‐CD80/86), BCs (CD40‐CD40LG) and myeloid cells (CSF2‐CSF2RA). The interactions of CSF2 and its receptors between Th17 cells and myeloid cell subsets, were specifically presented in IRBP_1−20_‐immunized EAU mice, but not in blank and control mice, and were enhanced following SL. Overall, SL amplified autoimmune responses following EAU establishment, especially in Th17 cells, which may contribute to heightened EAU symptoms.

Since pathological Th17 cells are crucial for AU development,^17^ understanding how Th17 cells become pathogenic is essential for treating autoimmune disorders. Although previous studies have found success,^67^ few have considered SL as an underlying factor or its mechanisms. Th17‐cell pathogenicity is manifested through cytokine secretion, such as GM‐CSF and IL‐17, which then bind to receptors on other cells, such as myeloid cells, resulting in cell differentiation and activation. In scRNA‐seq, we found that SL promoted the differentiation and activation of Th17 and myeloid cells, increased GM‐CSF level in Th17 cells and increased GM‐CSF receptor expression in myeloid cells. We next validated that the increased Th17‐derived GM‐CSF promoted IL‐23 secretion by CD11C^+^ cells, and targeting GM‐CSF could inhibit the CD11C^+^ cells infiltration in EAU mice. These results suggest that SL promotes myeloid cells activation through the GM‐CSF–myeloid–IL‐23 signalling pathway. Notably, we found SL also upregulated the IL‐23 signalling pathway in Th17 cells after EAU induction in scRNA‐seq data. GM‐CSF is a downstream cytokine of IL‐23/L‐23R signalling, and myeloid‐secreted IL‐23 promotes Th17 pathogenicity and further GM‐CSF secretion.^38^ Thus, we validated the increased ratio of Th17 cells expressing IL‐23R in SU group, and their IL‐23R and GM‐CSF levels increased after IL‐23 stimulation. Moreover, GM‐CSF was reported to promote CCR2^+^ monocyte activation and initiate monocyte expressing IL‐1β, thereby causing tissue inflammation.^57^ CCR2 is an inflammatory marker of myeloid cells, which can induced by the renal tubular cell‐derived GM‐CSF and thereby promoted the CCL‐2/CCR2‐mediated fibrosis and inflammation.^68^ We found that SL increased GM‐CSF receptors, CCR2 and IL‐1β in myeloid cells, and validated that Th17‐derived GM‐CSF could promote CCR2 expression, suggesting that Th17‐derived GM‐CSF facilitates the secretion of cytokine (IL‐23) and cell migration (CCR2) in myeloid cells. Thus, GM‐CSF and IL‐23 enhanced Th17 pathogenicity by forming a positive feedback loop, which contributed to the aggravated EAU after SL. Moreover, we demonstrated that anti‐GM‐CSF therapy exhibited therapeutic effects on SL‐induced EAU aggravation by inhibiting the positive feedback mechanism. Furthermore, we validated that the concentrations of GM‐CSF, IL‐23 and IL‐17A were elevated in patients with SL‐induced recurrent AU and decreased in patients receiving conventional therapy. Overall, we demonstrated that SL increases GM‐CSF levels, promotes Th17 pathogenicity by the IL‐23–Th17–GM‐CSF feedback mechanism and identified GM‐CSF as a target for treating SL‐induced relapse of AU.

By simulating the condition of SL in humans and establishing the sleep deprivation model in mice, we explored the impacts and mechanisms of SL on immune cells and autoimmunity. However, our study design still has several important limitations. On the one hand, our methodology of SL, especially in mice, did not consider the influence of circadian rhythm differences. The circadian system of mammals is regulated by the circadian clock, a transcriptional‐translation feedback loop composed of master clock genes.^69^ In mice, light is the most common environmental factor influencing the change of the circadian rhythm.^70^ Three rounds of sleep deprivation during our establishment of the SL model would probably disturb the balance of the circadian clock in mice. Nevertheless, in our study design, the SL mice were kept awake by forced activity induced by the intermittent rotation of slowly rotating drums programmed on a repeating cycle without changing their light condition. We believe that the impact of confounding factors during the study can be minimised. On the other hand, the size of samples included in the scRNA‐seq study is relatively small due to the high cost and peculiarity of single‐cell technologies.

## 4 CONCLUSIONS

In summary, we used high‐dimensional analysis to construct the first comprehensive immune cell atlas to explore the impact of SL on autoimmunity both in an EAU mouse model and in patients with AU. SL primarily affected CD4^+^ TCs and myeloid cells, and enhanced the interactions between Th17 and myeloid cell. Importantly, we revealed that SL promotes GM‐CSF secretion and the IL‐23–Th17–GM‐CSF feedback mechanism to enhance Th17 pathogenicity. Furthermore, targeted inhibition of GM‐CSF blocked EAU development, identifying GM‐CSF as a target to treat SL‐induced relapse of AU. This study provides a unique view of the immune mechanisms underlying the SL‐induced autoimmune predisposition, which could serve as a resource for future clinical research.

## 5 MATERIALS AND METHODS

### 5.1 Human subjects’ enrolment

Six healthy individuals were recruited for the sleep‐loss study (Supporting Information Table S1). The inclusionary criteria included age range from 35 to 55 years; physical and psychological health; regular sleep habits with approximately 8 h of sleep (22.00–06.00). Exclusion criteria included obesity, medication, smoking, binge drinking, excessive caffeine use (>three cups per day) and sleep or circadian disorders.

In addition, 12 AU patients under different stages were also recruited. The AU patients were diagnosed by referring to the symptoms, vitreous and anterior chamber inflammation, according to the Nussenblat scale and Standardization of Uveitis Nomenclature Working Group.^71^ AU patients in the initial stage refer to patients who have had disease onset within 1 month with obvious symptoms of uveitis. Patients with SL‐induced recurrent uveitis are defined as uveitis patients who displayed stable disease condition for more than 3 months, but appeared to have obvious relapsing symptoms including redness and pain in the eye, vision loss, anterior chamber flash and cell rating increased by two grades with or without macular oedema after 1 to 3 days of working through the night, despite excluding factors such as diet, medication and emotion. As for patients with SL‐induced recurrent uveitis, we have treated them with conventional therapy. After treatment, the patients recuperated to stability for at least 1 month, characterised by restored vision and resumed the prescription of steroids at maintenance dosage. Medical records of patients are provided in Supporting Information Table S2.

### 5.2 Study protocol of SL

In the screening stage, subjects were asked to stay in the laboratory of the Zhongshan Ophthalmic Centre for a habituation night to ensure their eligibility and adapt to the environment. Next, subjects were assigned to obey a strict sleep‐wake schedule (22:00‐06:00) in a fortnight by checking the sleep logs. After one habituation day (day 0), all participants have gone through two conditions (sleep regularly on day 1 and SL for 24 h on day 2). On one day 1, they slept for 8 h (22:00‐06:00) in the laboratory, and from 06:00 to 07:00, they took the 24 h periods of SL (day 2). Laboratory environmental conditions were highly controlled including ambient light and temperature. Under monitoring of research staff, subjects went outdoors at least three times in the daytime (06:00‐18:00) to parallel normal behaviour. Exercising or strenuous activities were restricted for participants. Non‐vigorous activities, like reading, watching television, browsing the internet, consuming food and drink, were permitted to be done in the given time (6:00‐22:00). To ensure wakefulness, participants were not permitted to close their eyes during day 2 under the persistent surveillance of three study staff. After completement of the study, mental states of the participants were assessed and displayed anxious and sleepy subjectively, suggesting that human did not easily habituate to SL.

After half an hour of sitting, blood samples from participants were collected at 06:00‐07:00 on day 2, the time after 24 h of SL. PBMCs were isolated, and the cell viability in all samples exceeded 90%, referring to greater than 1 × 10^7^ viable cells. In the stimulation group, the cells were stimulated by phorbol myristate acetate with a concentration of 50 ng/mL, ionomycin with a concentration of 500 ng/mL and brefeldin A (Sigma) with a concentration of 1 μg/mL at 37°C for 4 h. Then, PBMCs with or without antigen‐independent stimulation were obtained to analyse the expression of proteins among various lineage‐, activation‐ and migration‐related surface markers, as well as the simultaneous analysis of secreted cytokines under the single‐cell resolution (n = 24; Supporting Information Table S3).

### 5.3 Mice

We purchase SPF grade C57BL/6J mice (female, 6−8 weeks old, 20−25 g) from Guangzhou Animal Experiment Company. Mice were kept on a standard 12 h:12 h light‐dark cycle (lights on at 6: 00 AM) with food and water supplied ad libitum. All procedures were performed compliant to the ARVO Animal Statement for the Use of Animals in Ophthalmic and Vision Research.

### 5.4 Induction of EAU

To effectively induce EAU production, we injected mice subcutaneously with a 1:1 mixture of 200 µg IRBP_1−20_ (GL Biochem, Shanghai, P. R. China) and complete Freund's adjuvant (Difco, Detroit, MI, USA) including 2.5 mg/mL of *Mycobacterium tuberculosis* strain H37Ra (BD Difco, San Jose, CA, USA). After immunization, 0.25 μg of PTX (List Biological Laboratories, Campbell, California, USA) was injected intraperitoneally on days 0 and 2.

### 5.5 Experimental design of SL in mice

After a week of acclimation, the mice were randomly allocated to one of the five groups: (1) normal blank group, (2) normal mice undergoing SL (SL group), (3) IRBP_1−20_‐immunized EAU mice (EAU group), (4) IRBP_1−20_‐immunized EAU mice undergoing SL (SU) and (5) mice model without IRBP_1−20_ (control group). For SU and SL groups, SL measures were administered. The specific scheme is as follows: the mice should first adapt to the equipment environment to ensure regular sleep for 1 week. Three cycles of SL were performed during the 14‐day of EAU induction. During each cycle, mice would experience a 48‐h continuous sleep deprivation period, which began at 16:00 on the first day to 16:00 on the third day (Supporting Information Figure S2A). The sleep‐restricted mice were forced to keep awake by the intermittent rotation of slowly rotating drums (40 cm in diameter, rotation speed 15 rpm/min) programmed on a repeating cycle of 10 s on (approximately 2.5 rpm) and 1‐min off schedule. Mice of the five groups were kept on a standard 12 h:12 h light‐dark cycle with the supply of food and water ad libitum. At the end of EAU induction and SL induction, the retina and CDLNs were collected for further experiments.

### 5.6 Treatment with anti‐GM‐CSF in vivo

A total of 10 mg/kg recombinant anti‐GM‐CSF antibodies (#505417, BioLegend, San Diego, USA) or PBS was injected intraperitoneally into SU mice on days 1, 0, 2, 5 and 10 after EAU immunization. On day 14, tissue samples were taken for subsequent histological and flow cytometric analysis.

### 5.7 Clinical and histopathologic assessment

Fourteen days after immunization, fundus photography was performed with a Micron IV retinal imaging microscope (PHOENIX, USA). According to the condition of the fundus, we made a clinical score (0–4 points). For histopathological scores, the eyeballs were immersed in 4% neutral formalin buffer for 24 h. Histopathologic examination was performed with standard haematoxylin and eosin staining. The severity of EAU (0–4 points) refers to previously published criteria.^51^

### 5.8 Preparation of cell suspension of CDLNs

On day 14 after immunization, the CDLNs of blank, SL, control, EAU and SU mice were eviscerated, and the cell suspensions were acquired by grinding the organs through nylon mesh to get the cell pellet. Then, cells were prepared for subsequent studies. For scRNA‐seq, one sample has merged CDLNs cells from three mice from the same group to ensure sufficient number of cells for sequencing.

### 5.9 Treatment of CDLNs cells in vitro

The cells were cultured in 96‐well plates and stimulated by IRBP_1−20_ (20 μg/mL) with or without IL‐23 (20 ng/mL, PeproTech, USA), or anti‐IL‐23 (10 μg/mL, #513802, BioLegend) at 37°C for 72 h in a humidified incubator with 5% CO_2_.

### 5.10 Flow cytometry analysis

Cells from the retina and CDLNs were collected, washed with live or dead dye (#423105) and PBS and stained with the following antibodies: The surface markers included: CD4 (PerCP/Cy5.5, #100434), IL‐23R (BV421, #150907) CD11C (PerCP/Cy5.5, #117328). For intracellular cytokine staining, the cells were stimulated with 50 ng/mL of phorbol myristate acetate, 500 ng/mL of ionomycin and 1 μg/mL brefeldin A (Sigma) at 37°C for 5 h. After fixation and permeabilisation, cells were stained with antibodies: IFN‐γ (PE, #505808), IL‐17A (AF647, #506912) and GM‐CSF (PE‐Cy7, #505411). For human PBMCs, the surface markers included: CD3 (PE/Cy7, #300419), CD8 (BV605, #344742), CD19 (BV786, #302240) and CD14 (PE/Dazzle 594, #301852). The intracellular antibodies included: IL‐17A (Pacific Blue, #512311), GM‐CSF (PerCP/Cy5.5, #502312), IL‐23 p40 (FITC, #501804) (BioLegend). The cells were measured and were analysed with FlowJo software (version 10.0.7, USA).

### 5.11 Co‐cultivation of CD11C^+^ cells with CD4^+^CCR6^+^CXCR3^‐^ TCs

Th17 cells (CD4^+^CCR6^+^CXCR3^‐^ TCs) and CD11C^+^ cells were isolated by fluorescent cell sorting. Then, sorted CD4^+^CCR6^+^CXCR3^‐^TCs and CD11C^+^ cells were co‐cultured at a ratio of 2:1 with or without anti‐GM‐CSF (10 μg/mL) or GM‐CSF (20 ng/mL, PeproTech) for 48 h.

### 5.12 IRBP antibody measurement

On the 14th day of immunization, serum from mice in different groups was collected. For the detection of IgG antibody against IRBP_1−20_, the wells were coated with 1 μg/mL of IRBP_1−20_ at 4°C for a night. Serum samples were added into the wells and incubated at 37°C for 1.5 h. After washing the wells, the bound antibody was detected with goat anti‐mouse IgG for 1.5 h (Southern Biotechnology, Birmingham, AL, USA). Finally, read the absorbance at 450 nm and calculate the mean optical density for each group.

### 5.13 ELISA

After obtaining the PBMCs of healthy subjects and patients at different stages (initial onset, post‐treatment and recurrence due to SL), we conducted the following ELISA kit detection, obeying the manufacturer's instructions: human IL‐17A ELISA Kit (#88‐7176‐22), human IL‐23 ELISA Kit (#88‐7237‐22) and human GM‐CSF ELISA Kit (#88‐8337‐88). The supernatants from cell co‐culture medium were determined using the mouse IL‐23 ELISA Kit (#BMS6017). The serum from mice were determined using the mouse IL‐23 ELISA Kit (#BMS6017) and GM‐CSF mouse ELISA Kit (#BMS612) (Thermo Fisher Scientific).

### 5.14 RNA isolation and real‐time quantitative PCR

Total RNA was extracted with Trizol, and then level was measured with a NanoDrop spectrophotometer. Next, cDNAs were synthesised using the PrimeScript™ RT Master Mix. Real‐time quantitative PCR was performed using SYBR Premix Ex TaqTM II (TaKaRa Bio Inc.). Primer sequences were as follows: mice β‐actin (Forward: 5′‐GAGCGCAAGTACTCTGTGTG‐3′, Reverse: 5′‐AGCTCAGTAACAGTCCGCC‐3′); mice Ccr2 (Forward: 5′‐TCATCCACGGCATACTATCAA‐3′, Reverse: 5′‐TATTCCCAAAGACCCACTCAT‐3′). The relative mRNA levels of Ccr2 were determined using the 2–ΔΔ*Ct* method based on the control level of β‐actin mRNA.

### 5.15 Adoptive transfer experiment

The CD4^+^ TCs were separated from the CDLNs and spleens of EAU and SU mice on day 14 after immunization and stimulated by IRBP_1−20_ (20 μg/mL) for 3 days. The isolated cells from SU mice were also cultured in the presence of anti‐GM‐CSF (10 μg/mL) or vehicle. A total of 2 × 10^7^ cells were injected into each normal C57BL/6J mouse.

### 5.16 Mass cytometry

#### 5.16.1 Antibodies and reagents

The antibodies are given in Supporting Information Table S3. They were pre‐conjugated antibodies that were purchased from a commercial company or conjugated manually (Fluidigm). The following steps were adapted from the published article.^26^

#### 5.16.2 Live‐cell barcoding and antibody staining

A live‐cell barcoding method was used to decrease inter‐sample staining variability. The barcoded and combined samples were stained with 0.5 μmol/L cisplatin‐195pt. The cells were re‐suspended in pre‐cooled Maxpar Cell Staining Buffer and washed with PBS/bovine serum albumin. For surface staining, the cells were added to a surface antibody mixture and incubated at 37°C for 30 min. The samples were stored in 2% formaldehyde in PBS at 4°C overnight. Then, the cells were washed and re‐suspended in intracellular antibodies mixture for 1 h at 4°C. The samples were then added to iridium intercalator solution (Fluidigm) overnight.

#### 5.16.3 Mass cytometry acquiring and processing

The data were obtained from a SuperSampler fluidics CyTOF2 system (Victorian Airships, Alamo, CA), and then normalised with Helios normalizer software (version 6.7.1016). Based on event length, live cell (195Pt), and DNA (191Ir and 193Ir) channels, Cytobank was used to sequentially remove calibration beads, dead cells, debris and export FCS files. Specially, we randomly selected 10 000 cells per sample for downstream analysis. The CATALYST was used to integrate data. After dimension reduction and clustering, the FlowSOM‐based nodes were performed to identify 15 populations. The detailed cell numbers are given in Supporting Information Table S4.

### 5.17 scRNA‐seq

#### 5.17.1 scRNA‐seq data alignment, processing and sample aggregation

Using the Chromium Single Cell 5′ Library (10× Genomics, Genomics chromium platform Illumina NovaSeq 6000), Chip Kit (10× Genomics) and Gel Bead and Multiplex Kit, cells were transformed into barcoded libraries. The library's quality was checked by FastQC software. The CellRanger software, in which the count pipeline was used to demultiplex and barcode the sequences, was applied to preliminary process the Sequencing data. The Seurat package (version 4.0.5) was applied to perform the subsequent analysis with the default parameters. Finally, a total of 96 465 cells (blank, 12 222 cells; SL, 14 308 cells; control, 12 074 cells; EAU, 12 313 cells; SU, 9431 cells) were analysed after quality control (mitochondrial gene ratio < 15%, detected genes > 600).

#### 5.17.2 Differential expression analysis

Differential expression analysis for each subset between different groups (SL/blank, control/blank, EAU/control, EAU/blank, SU/SL and SU/EAU) was performed and screened (|LogFC| > 0.25, *p*‐value < .05) using the Wilcoxon rank‐sum test as implemented in the ‘FindAllMarkers’ function of the Seurat package (version 4.0.5).

#### 5.17.3 Gene functional annotation

GO biological process and pathway analyses were conducted in the Metascape webtool (www.metascape.org).^72^ The SL‐ or EAU‐related top 10 of 30 terms were visualised by the ggplot2 R package.

#### 5.17.4 Determination of cell–cell interactions

We predicted cell–cell interactions among different cells by using CellPhoneDB and CellChat R packages. The method in CellPhoneDB prioritises cell type‐specific expressing ligand–receptor interactions so as to identify the most significant interactions between cell types. Therefore, the cluster labels of each cell were randomly permuting 1000 times during the pairwise cluster–cluster interaction analyses. During each permutation, the package calculates the mean of the average expression level of receptor in one cluster and the ligand in its interacting cluster, with a null distribution deriving from each receptor–ligand pair in every cell–cell interaction. The calculation of *p*‐value is based on which proportion of the means is ‘as or more extreme’ than the actual one. All the interacting partners have carried on the significant mean calculation. The cell–cell interaction was considered significant in case of *p* value < .05, and the mean value was kept under this condition. Based on the analysis of CellPhoneDB, we analyse and visualise the differences in intracellular interaction among the five groups. In addition, CellChat was applied with default parameters to analyse and visualise signalling pathway networks among the five groups.

### 5.18 Statistics and reproducibility

In CyTOF analysis, the cell percentages between the post‐SL and pre‐SL groups were compared using two‐tailed paired *t*‐tests. For comparing changes in marker expression between human post‐SL and pre‐SL groups, adjusted *p* values were calculated using the ‘diffcyt‐DS‐GLMM’ method as implemented in the ‘diffcyt'function of diffcyt R package considering the samples pairing before and after SL. In scRNA‐seq, to compare the expression of genes between two groups, two‐sided Wilcoxon test, which was implemented in the function ‘compare_means’ of ggpubr R package, was used to calculate *p* value. When calculating the GO biological process and pathway terms, *p* values were calculated by a hypergeometric test in Metascape webtool. The scores between EAU and SU were compared using two‐tailed unpaired Student's *t*‐test. For the statistical analysis of variables in multiple groups, one‐way ANOVA with a Bonferroni post‐test was used. Finally, the analysis and visualization were performed using GraphPad Prism (version 8.0.2; GraphPad Software Inc.). Each group contains five or six mice, and the values represent the mean ± SD from three independent experiments. ns, not significant, *p < .05, **p < .01, ***p < .001, ****p < .0001.

## CONFLICTS OF INTEREST STATEMENT

The authors declare no conflict of interest.

## Supporting information

Supporting Information

Supporting Information

## Data Availability

The scRNA‐seq data of mice CDLNs is stored in the Genome Sequence Archive in BIG Data Center (https://bigd.big.ac.cn/gsa/), under the GSA Accession No. CRA008342, and the GSA Accession No. CRA003777.
