# Supplementary material for: Sleep loss potentiates Th17‐cell pathogenicity and promotes autoimmune uveitis
Source: Clin Transl Med. 2023 May 2;13(5):e1250. doi: 10.1002/ctm2.1250 (PMC10154899; doi:10.1002/ctm2.1250)

**Supplementary Figure and Legend**

**Figure S1. CyTOF validated the human blood immune dysregulation after SL.**

**A.** t-SNE plot showing the human blood immune subpopulations in CyTOF.

**B.** t-SNE plot showing the human blood immune subpopulations among 4 groups.

**C.** The heatmap showing the relative levels of cytokines and markers in CD4+ TCs under unstimulated condition. Color scheme is based on z-score distribution from min (white) to max (red). Significance was determined using the “diffcyt-DS-GLMM” method as implemented in the “diffcyt” function of diffcyt R package; ns, not signiﬁcant, *P < 0.05, **P < 0.01, ****P < 0.0001.

**D.** The violin plot showing the expression of CXCR5, CD45RO, and CD57 in CD4+ TCs under stimulated condition between preSL and postSL groups.

**E.** The percentage of IL-1β+ cells in CD3+CD4+ T cells measured by CyTOF. [Scatter](javascript:;) charts show the changes between the two groups derived from CyTOF data (n = 6/group). Significance was determined using two-tailed paired t-test; *P < 0.05.

The full names of the subsets are as follows: CD4NA, CD4+ naive T cell; CD4TCM, central memory CD4+ T cell; CD4Treg, regulatory CD4+ T cell; CD4TEM, effector memory CD4+ T cell; CD4CTL, cytotoxic CD4+ T cell; CD8NA, CD8+ naive T cell; CD8TEM, effector memory CD8+ T cell; CD8CTL, cytotoxic CD8+ T cell; Undefined, undefined T cells; NK, natural killer cell; BC, B cell; CMC, classical monocyte; NMC, nonclassical monocyte; CDC, conventional dendritic cell; PDC, plasmacytoid dendritic cell.

**Figure S2. SL induced immune dysregulation in mice LNs.**

**A.** Schematic of the experimental design for the induction of sleep-loss model and EAU model, and subsequent scRNA-seq processes.

**B.** t-SNE plot showing the immune subpopulations of CDLNs in scRNA-seq.

**C.** The heatmap showing scaled expression of discriminative gene sets for each subpopulation. Color scheme is based on z-score distribution from min (blue) to max (red).

**D.** The pie chart (left) and histogram (right) showing the relative percentage and number of immune subpopulations between Blank and SL groups.

**E.** Representative GO biological process and pathways enriched in upregulated SL-DEGs among immune subpopulations.

**F.** Venn diagram showing the interactions of upregulated SL-DEGs among immune subpopulations.

The full names of the subsets are as follows: CD4, CD4+ T cell; CYTO, cytotoxic cell including CD8+ T and NK cell; MITO, mitotic T cell; BC, B cell; TBC, T and B cell; PDC, plasmacytoid dendritic cell.

**Figure S3. SL induced autoimmune-associated changes in mice LNs.**

**A-C.** BC (**A**), CYTO (**B**), and myeloid cells (**C**) were re-clustered and identified classical subsets (top) based on the scaled expression heatmap of discriminative gene for each cluster (bottom). Color scheme is based on z-score distribution from min (blue) to max (red).

**D.** The heatmap showing the relative levels of marker genes in CD4+ T cell subsets. Color scheme is based on z-score distribution from min (white) to max (red).

**E.** The pie chart showing the relative percentage of CD4+ TC subsets between Blank and SL groups.

**F.** t-SNE plot showing the expression of *Ifng* in CD4+ TC in scRNA-seq data.

**G.** Volcano plot showing the upregulated or downregulated SL-DEGs in Treg.

**H.** The rose diagram showing the number of DEGs in SL/Blank comparison among myeloid cell subsets.

The full names of the subsets are as follows: NBC, naive B cell; NAIBC, naive Isg15+ B cell; MBC, memory B cell; PC, plasma cell; ABC, age/autoimmune-associated B cell; GBC, germinal B cell; CD4NA, CD4+ naive T cell; CD4NAI, CD4+ naive Isg15+ T cell; Treg, regulatory CD4+ T cell; Th1, T helper 1 cell; Th17, T helper 17 cell; TFH, Bcl6^high^ S1pr1^-^ T follicular helper cells; pre-TFH, Bcl6^med^ S1pr1^+^ TFH precursor; CD8NA, CD8+ naive T cell; CD8NAI, CD8+ naive Isg15+ T cell; CD8CTL, cytotoxic CD8+ T cell; NK, natural killer cell; CDC, conventional dendritic cell.

**Figure S4. SL promoted EAU development and enhanced the pathological immune response to EAU challenge.**

**A.** The representative fundus images of Blank, SL, and Ctrl mice at day 14.

**B.** Volcano plot showing the upregulated or downregulated DEGs in Ctrl/Blank comparison in all cells.

**C.** Volcano plot showing the upregulated or downregulated DEGs in EAU/Ctrl comparison in all cells.

**D.** Representative GO biological process and pathways enriched in upregulated DEGs in Ctrl/Blank, EAU/Ctrl, and EAU/Blank comparisons.

**Figure S5. SL resulted in aberrant intercellular interaction patterns during EAU.**

**A.** The heatmap showing the number of predicted cell-cell interaction among Blank, Ctrl, and SL groups.

**B.** The heatmap showing the relative levels of interaction among five groups. Color scheme is based on z-score distribution from min (white) to max (red). The grey indicates inexistence in this group.

**C.** Circle plot showing IL-2 signaling pathway network between SL and SU groups.

**Figure S6. Anti-GM-CSF treatment displayed rescue effects for the SL-induced aggravation of EAU.**

**A.** The gating strategies for retinal CD4+ and CD11C+ cells.

**B.** The gating strategies for CDLNs CD11C+ cells.

**C.** The gating strategies for CDLNs CD4+IL-17A+ Th17 cells.

**D.** Serum from EAU, SU, and SU+aGM groups were obtained after immunization at day 14. The column charts showing the serum level of IRBP_1-20_-specific IgG among three groups (n = 5/group). Data represented as mean ± SD. Significance was determined using one-way ANOVA; *P < 0.05. ****P < 0.0001.

**E.** The gating strategies for IL-23R and GM-CSF expression in CDLNs CD4+IL-17A+ Th17 cells.

**F.** After 48-h coculture of Th17 with CD11C+ cells with or without GM-CSF, the supernatant level of IL-23 was measured by ELISA. Data represented as mean ± SD. Significance was determined using one-way ANOVA; ***P < 0.001. ****P < 0.0001.

**Figure S7. SL increased Th17 pathogenicity by enhancing the IL-23–Th17–GM-CSF positive feedback mechanism.**

**A-B．**CD4+ T cells from EAU and SU groups cultured with IRBP_1-20_ alone or with IRBP_1-20_ plus with anti-IL-23 antibody for 72 h. The ﬂow cytometry histograms (left) and column charts (right) showing the percentage of CD4+IL-17A+ Th17 cells (**A**) and GM-CSF+ cells in CD4+IL-17A+ Th17 cells (**B**) (n = 5/group). Data represented as mean ± SD. Significance was determined using one-way ANOVA; ns, not signiﬁcant, **P < 0.01. ***P < 0.001. ****P < 0.0001.

**C.** The gating strategies for GM-CSF expression in human PBMCs Th17 cells.

**D.** The gating strategies for IL-23 expression in human PBMCs CD14+ monocytes.

**E-F.** PBMC were obtained from HC, SL, and AU patients under different stages. The ﬂow cytometry histograms (left) and column charts (right) showing the percentage of GM-CSF+ cells in Th17 cells (**E**) and IL-23+ cells in CD14+ monocytes (**F**) (n = 5/group). Data represented as mean ± SD. Significance was determined using one-way ANOVA; *P < 0.05. ***P < 0.001. ****P < 0.0001.

**Figure S8. The IL-23–Th17–GM-CSF positive feedback loop drive aggravated autoimmune uveitis after sleep loss.**

This diagram illustrates that sleep loss (SL) enhances Th17 pathogenicity and promotes autoimmune uveitis via the IL-23–Th17–GM-CSF positive feedback loop. SL increases GM-CSF levels in healthy individuals, patients with SL-induced recurrent uveitis, and mice with experimental autoimmune uveitis (EAU). SL enhances the interaction between Th17 and antigen presenting cells (APC). Th17-derived GM-CSF and APC-derived IL-23 construct a positive feedback loop to maintain Th17 pathogenicity. SL enhances Th17 pathogenicity through the IL-23–Th17–GM-CSF feedback mechanism, ultimately leading to AU development.


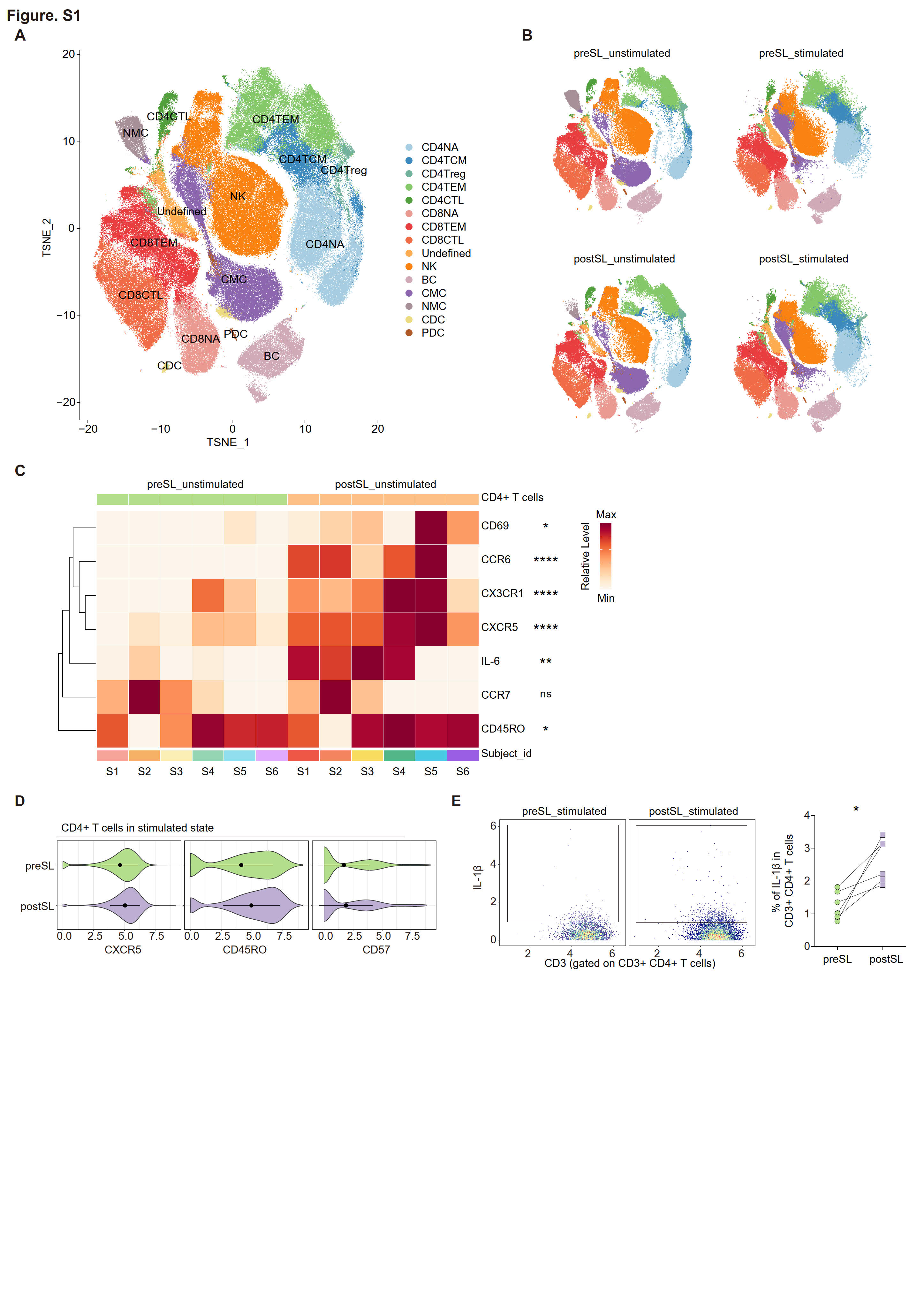


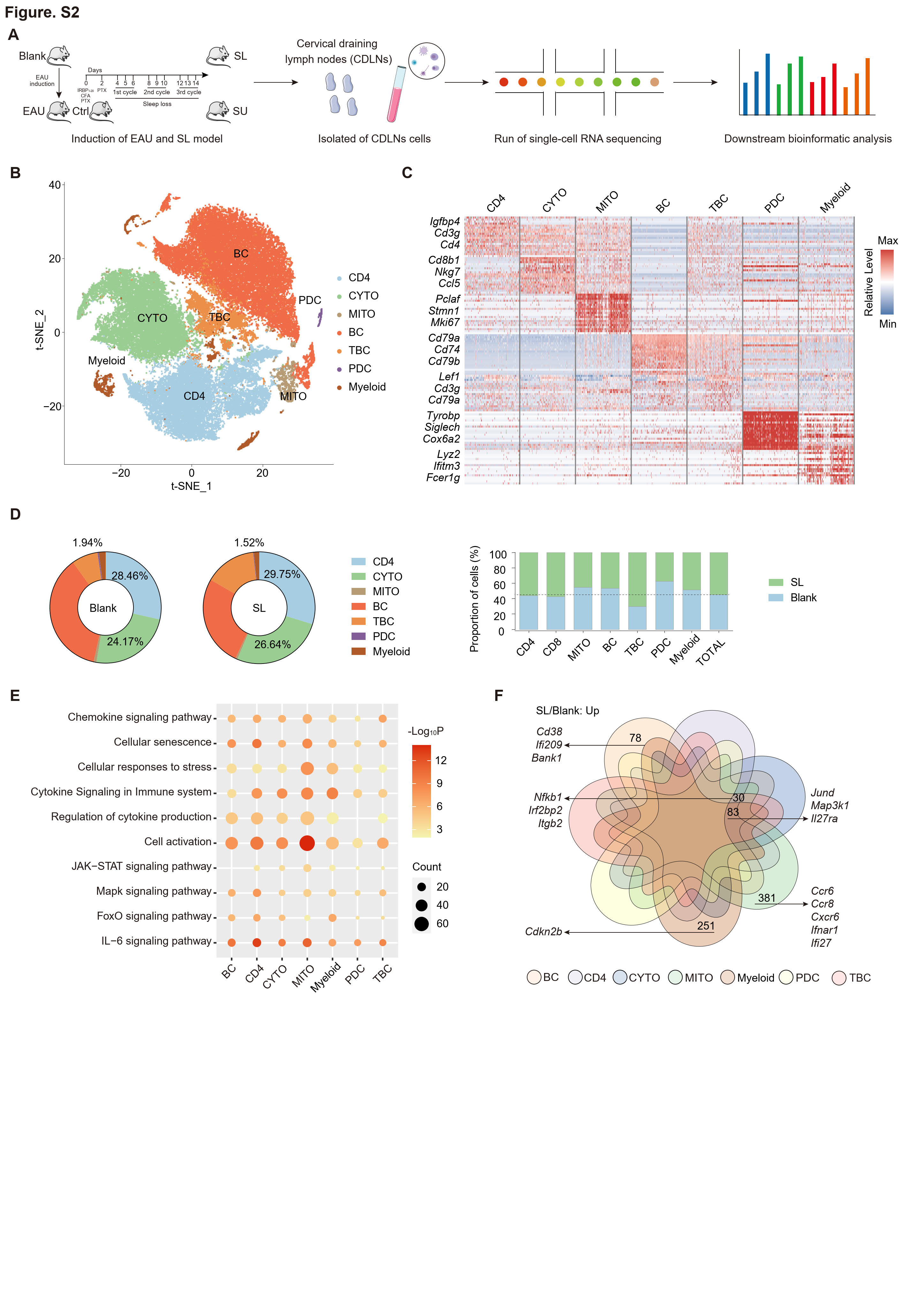


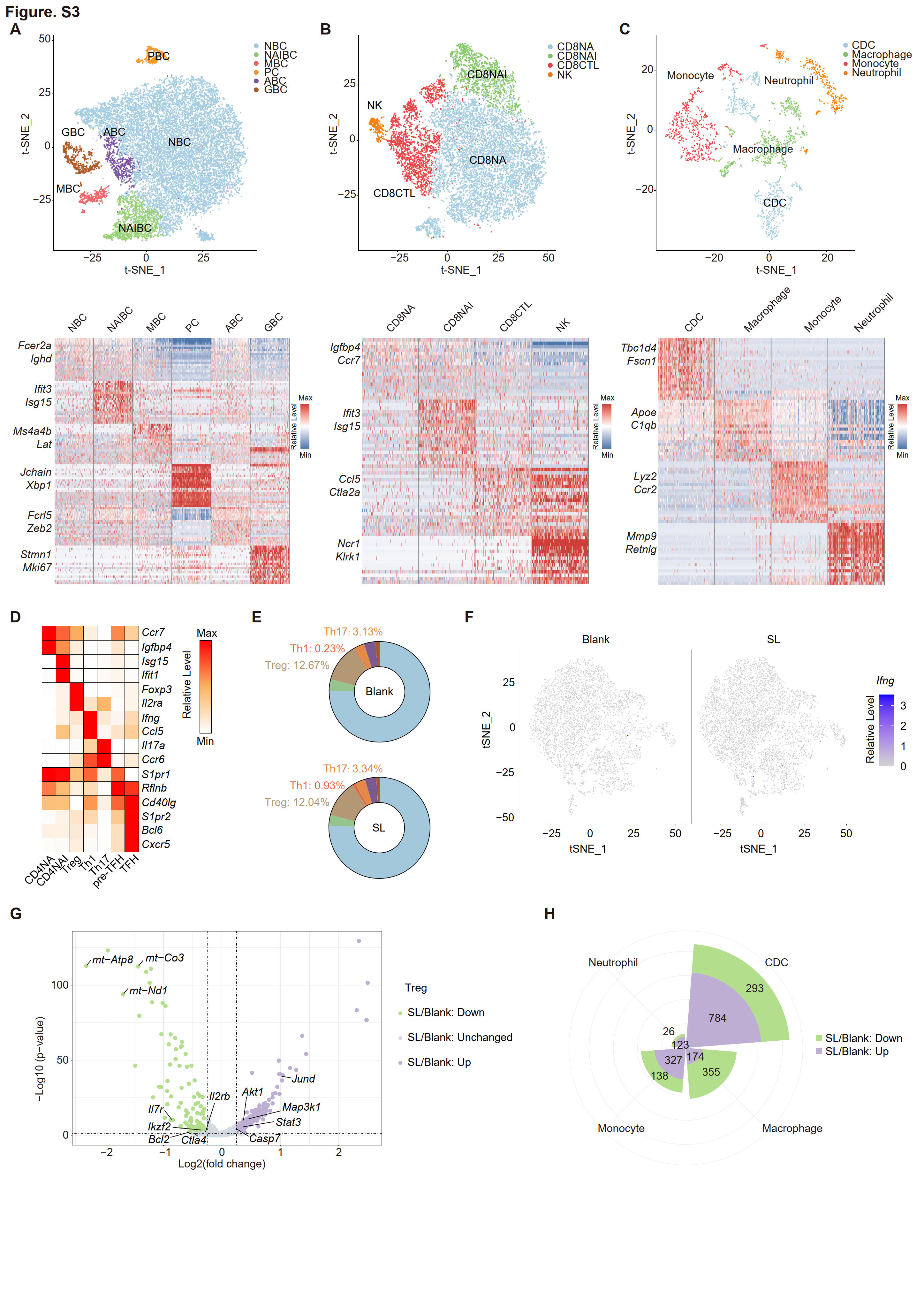


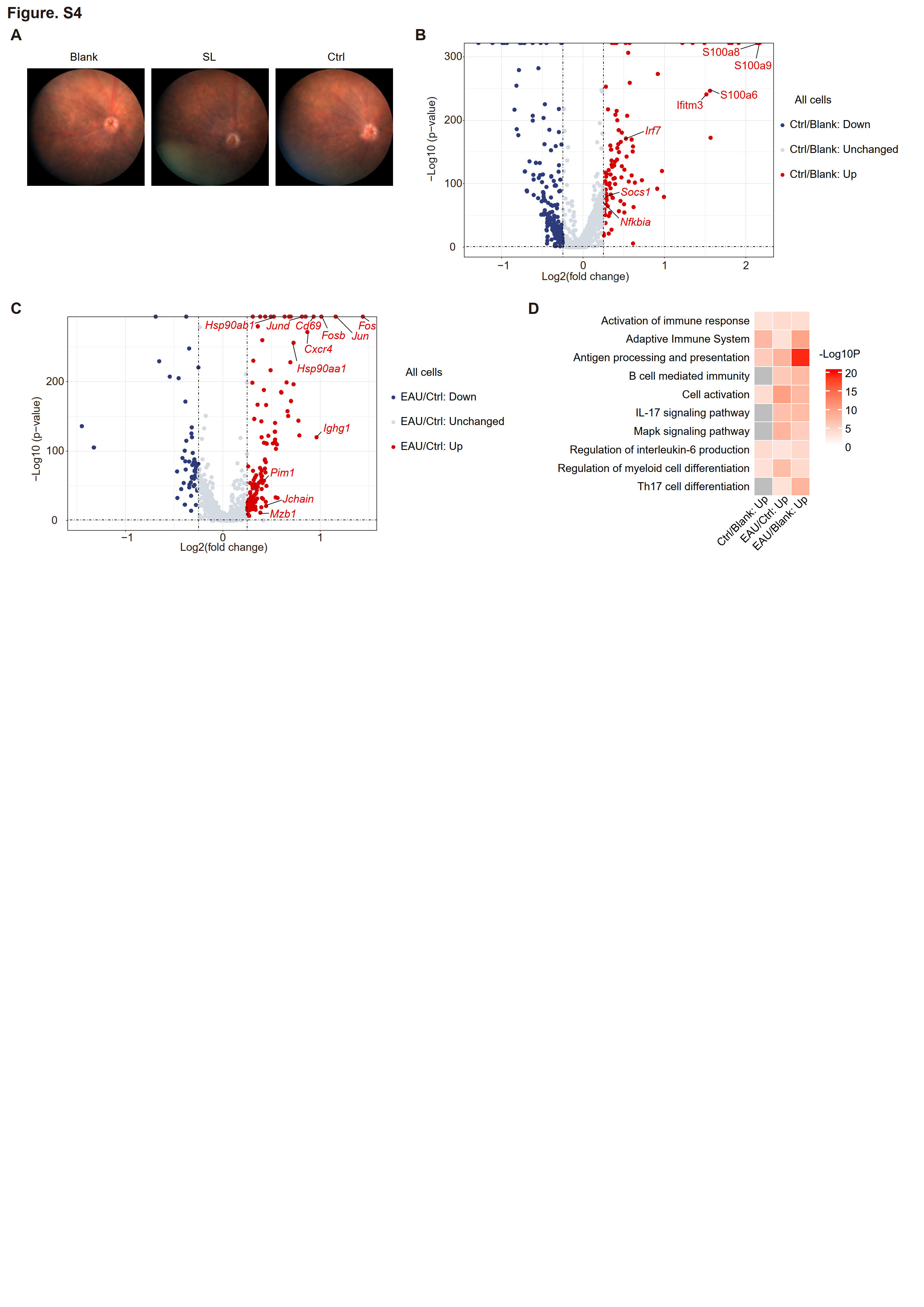


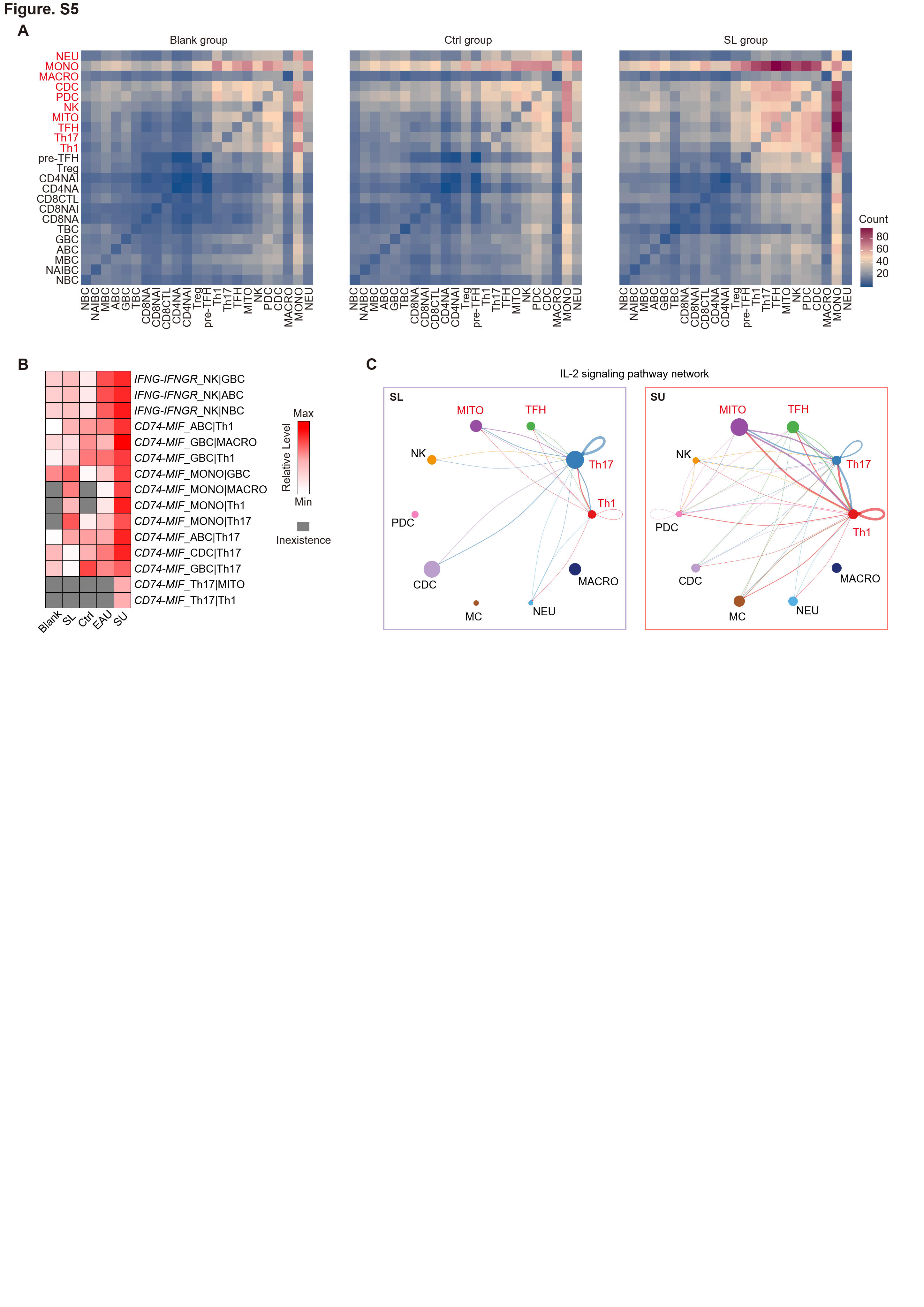


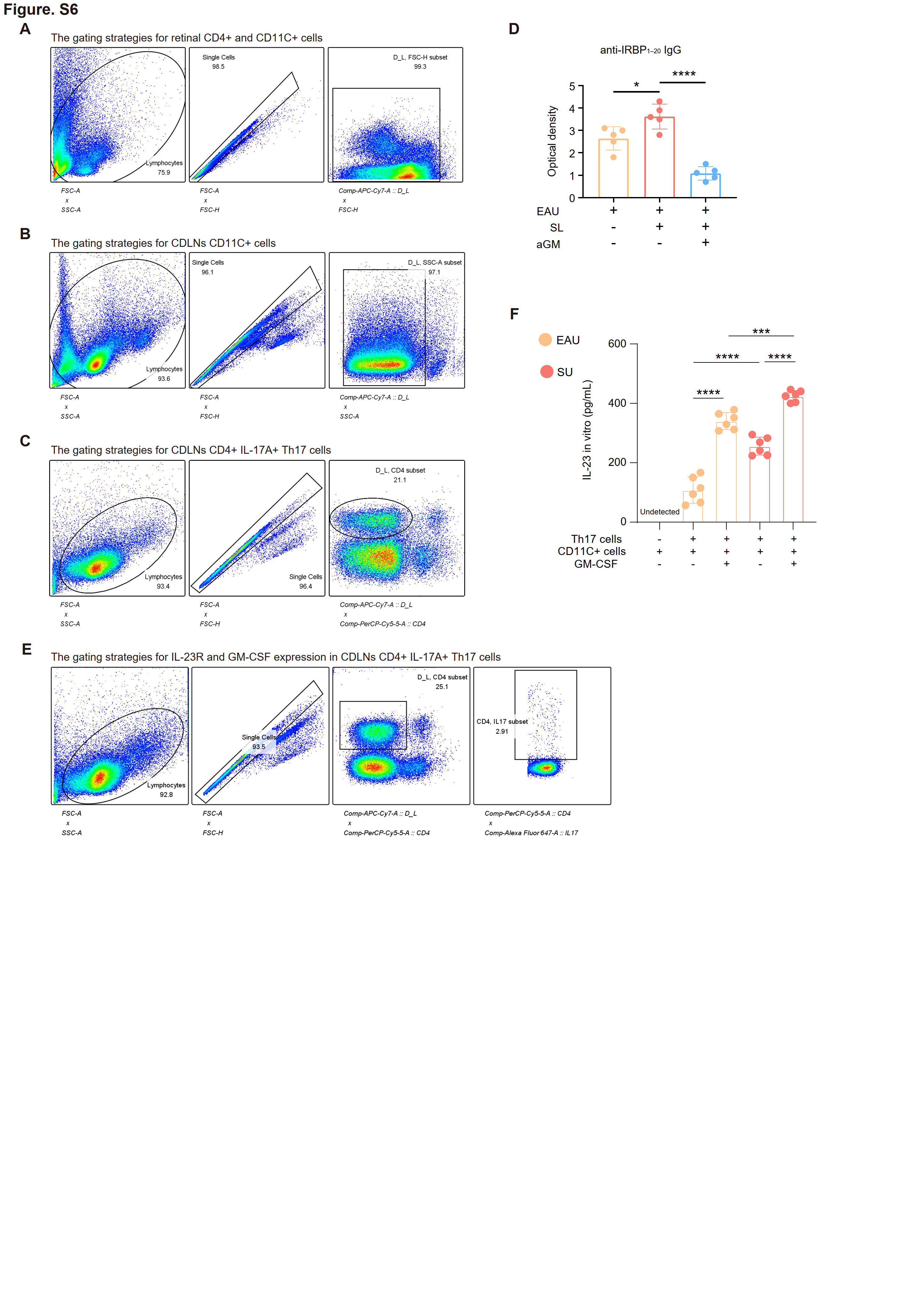


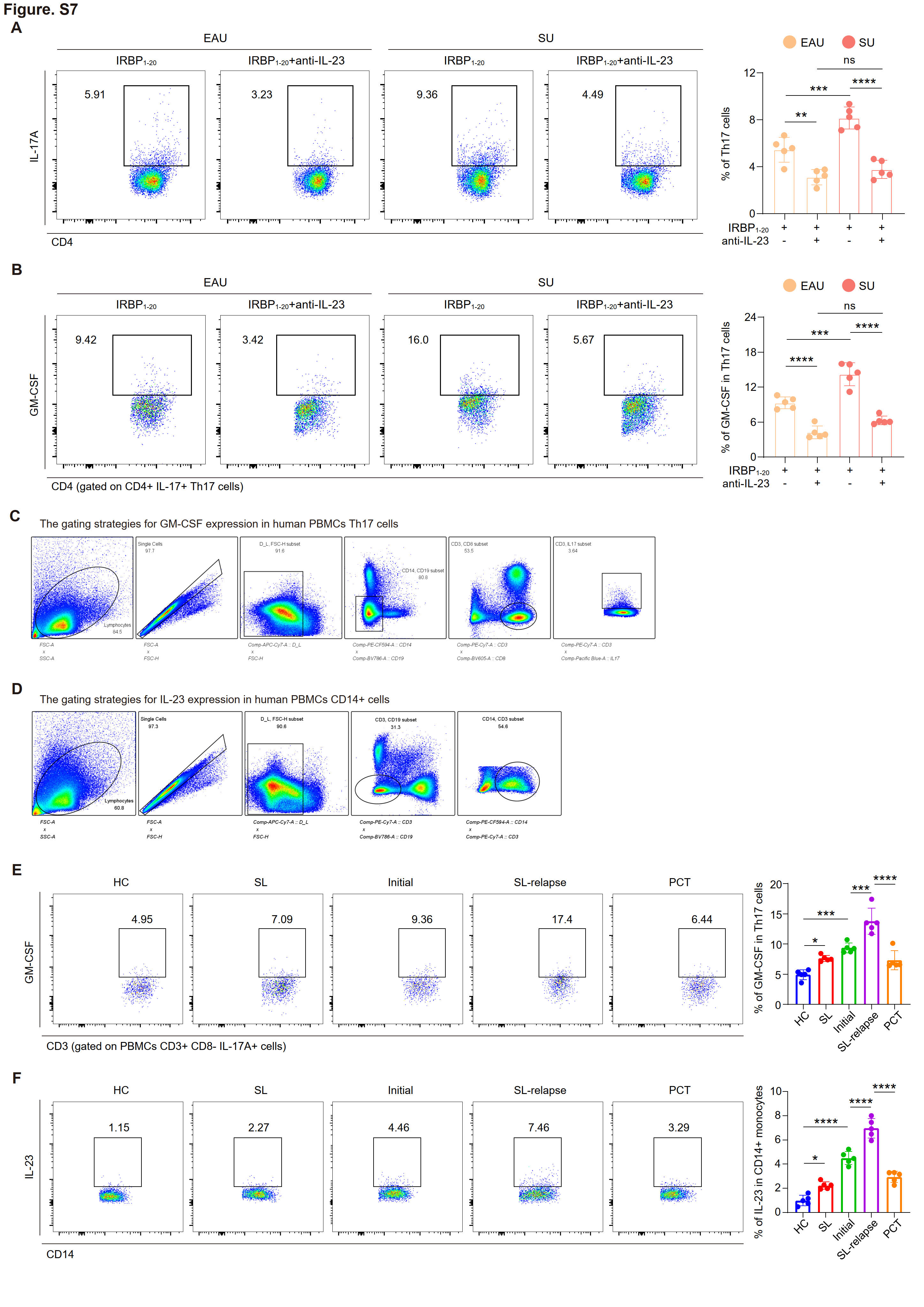


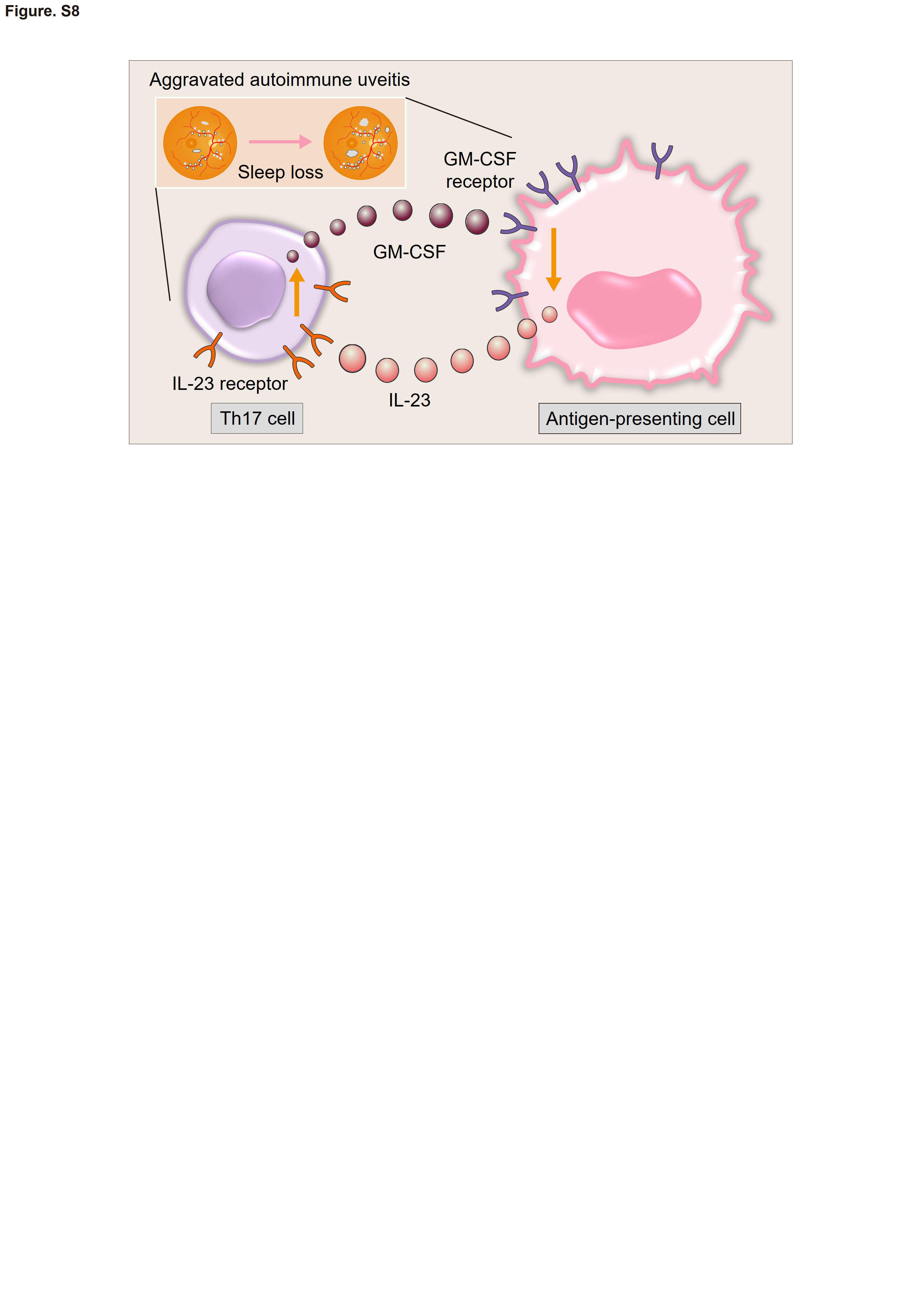

Supplement: Supplementary file 1 — Supporting Information [file CTM2-13-e1250-s001.docx]
